# Allele-Specific Virulence Attenuation of the *Pseudomonas syringae* HopZ1a Type III Effector via the *Arabidopsis* ZAR1 Resistance Protein

**DOI:** 10.1371/journal.pgen.1000894

**Published:** 2010-04-01

**Authors:** Jennifer D. Lewis, Ronald Wu, David S. Guttman, Darrell Desveaux

**Affiliations:** 1 Department of Cell and Systems Biology, University of Toronto, Toronto, Ontario, Canada; 2 Centre for the Analysis of Genome Evolution and Function, University of Toronto, Toronto, Ontario, Canada; The University of North Carolina at Chapel Hill, United States of America

## Abstract

Plant resistance (R) proteins provide a robust surveillance system to defend against potential pathogens. Despite their importance in plant innate immunity, relatively few of the ∼170 R proteins in *Arabidopsis* have well-characterized resistance specificity. In order to identify the R protein responsible for recognition of the *Pseudomonas syringae* type III secreted effector (T3SE) HopZ1a, we assembled an *Arabidopsis R* gene T–DNA Insertion Collection (ARTIC) from publicly available *Arabidopsis thaliana* insertion lines and screened it for plants lacking HopZ1a-induced immunity. This reverse genetic screen revealed that the *Arabidopsis* R protein HOPZ-ACTIVATED RESISTANCE 1 (ZAR1; At3g50950) is required for recognition of HopZ1a in *Arabidopsis*. ZAR1 belongs to the coiled-coil (CC) class of nucleotide binding site and leucine-rich repeat (NBS–LRR) containing R proteins; however, the ZAR1 CC domain phylogenetically clusters in a clade distinct from other related *Arabidopsis* R proteins. ZAR1–mediated immunity is independent of several genes required by other R protein signaling pathways, including *NDR1* and *RAR1*, suggesting that ZAR1 possesses distinct signaling requirements. The closely-related T3SE protein, HopZ1b, is still recognized by *zar1 Arabidopsis* plants indicating that *Arabidopsis* has evolved at least two independent R proteins to recognize the HopZ T3SE family. Also, in *Arabidopsis zar1* plants HopZ1a promotes *P. syringae* growth indicative of an ancestral virulence function for this T3SE prior to the evolution of recognition by the host resistance protein ZAR1. Our results demonstrate that the *Arabidopsis* resistance protein ZAR1 confers allele-specific recognition and virulence attenuation of the *Pseudomonas syringae* T3SE protein HopZ1a.

## Author Summary

*Pseudomonas syringae* is a model bacterial pathogen that can infect a broad range of plant species, including important crop plants, as well as the model plant *Arabidopsis thaliana*. *P. syringae* employs a specialized syringe-like structure called the type III secretion system to inject virulence proteins termed “effectors” directly into the cells of its plant host. In response, plants have evolved a surveillance system to recognize the presence of type III secreted effector (T3SE) proteins as a trigger for immunity. The sentinels of this surveillance system are termed resistance (R) proteins. Here we identify a new resistance protein, ZAR1, which recognizes the T3SE HopZ1a from *P. syringae*. HopZ1a is part of the important YopJ superfamily of T3SEs whose archetypical member, YopJ, is found in the causal agent of the bubonic plague, *Yersinia pestis*. We show that ZAR1–mediated immunity is independent of known *Arabidopsis* resistance-related genes suggesting that ZAR1 possesses novel signaling requirements. Interestingly, in *Arabidopsis* plants lacking ZAR1, HopZ1a enhances the virulence of *P. syringae* indicating that ZAR1 has evolved to recognize and attenuate an ancestral HopZ1a virulence function.

## Introduction

The retaliatory arms race between host and pathogen has molded the evolution of host immune responses and bacterial virulence strategies. The primary virulence mechanism of Gram-negative bacteria such as *Pseudomonas syringae* is the type III secretion system (T3SS) that allows for the translocation of type III secreted effector (T3SE) proteins directly into plant cells [1]. T3SEs may promote bacterial proliferation by manipulating host physiology or by suppressing host defenses [2]–[6]. However T3SEs can also betray the bacteria to the plant host by activating effector triggered immunity (ETI) [7]. ETI is a branch of plant immunity in which Resistance (R) proteins recognize specific effector proteins resulting in an effective immune response which is often accompanied by a rapid, localized cell death termed the hypersensitive response (HR) [8],[9]. Resistance proteins have been demonstrated to recognize T3SE proteins in two ways. In one case, the *Ralstonia solanacearum* T3SE PopP2 interacts directly with its cognate R protein RRS1-R [10]. As well, the *Xanthomonas campestris* T3SE AvrBs3 binds directly to the promoter of its cognate *R* gene *Bs3*, as *Bs3* has evolved to mimic virulence targets of AvrBs3 [11],[12]. In most cases however, the resistance protein indirectly recognizes the T3SE by interacting with a host target of the T3SE [9]. In the indirect mode of recognition, R proteins monitor a specific host T3SE target and ETI is initiated when this target is modified by the T3SE [9],[13]. Evolutionary pressure by pathogens has caused the expansion of several R protein families and the diversification of the signaling components which they employ [14].

R proteins are typically defined as having a nucleotide-binding-site (NBS) and leucine-rich-repeat (LRR) domain [15],[16]. In addition to the NBS-LRR domains, the N-terminal region is usually a coiled-coil (CC) domain or a TIR domain, named according to its homology to the *Drosophila* Toll and mammalian interleukin-1 receptors. Genetic studies of several *Arabidopsis R* genes have revealed important components of ETI signaling pathways [17]–[19]. ETI induced by CC-NBS-LRR class *R* proteins like RPS2, RPM1 and RPS5 requires NDR1, a membrane-localized glycosylphatidylinositol (GPI)-anchored protein [20]–[22]. TIR-NBS-LRR R proteins act through EDS1 and its interacting partner PAD4 and include R proteins recognizing effectors from *P. syringae* (RPS4) and the oomycete *Hyaloperonospora arabidopsidis* (RPP2, RPP4, RPP5, RPP21) [23]–[25]. R protein stability and accumulation can be mediated by SGT1 and its interacting partner RAR1, which was initially identified by its role in resistance to powdery mildews in barley [26]–[32]. In addition, salicylic acid (SA) and reactive oxygen species have been differentially implicated in the development of ETI and/or its corresponding HR [33],[34]. From the study of several *Arabidopsis* R proteins it is apparent that multiple ETI signaling pathways exist and more are likely to be uncovered as the ∼170 putative *Arabidopsis* R proteins are characterized further.

The HopZ family of *P. syringae* T3SE proteins is part of the larger YopJ superfamily with homologues in *Yersinia pestis* and *Xanthomonas* species [2],[35]. Evolutionary analyses demonstrated that the *P. syringae* pv. syringae (*Psy*) effector HopZ1a*_Psy_*_A2_ (formerly HopPsyH, hereafter HopZ1a) is most similar to the ancestral allele of the *P. syringae* HopZ family [35]. YopJ, the founding member of the HopZ/YopJ superfamily, has recently been shown to possess acetyltransferase activity [36]–[38]. YopJ acetylates serine and threonine residues of MAP kinase family members, which blocks the phosphorylation site needed for downstream immune signaling [37],[38]. Similar to YopJ, HopZ1a contains a canonical catalytic triad shared by proteases and acetyltransferases and requires the cysteine residue of this triad for enzymatic activity in a fluorescence-based protease assay [35]. HopZ1a induces defense responses characteristic of ETI in diverse plant hosts, including *Arabidopsis*, rice, sesame and soybean [35],[39]. The catalytic triad of HopZ1a is required for its recognition in *Arabidopsis*, indicating that it is recognized via its enzymatic activity [39]. Recognition of HopZ1a-induced immunity is induced independently of the characterized *Arabidopsis* R proteins RPM1, RPS2, RPS5 and RPS4 [39].

In this study, we demonstrate that the CC-NBS-LRR *R* gene, HOPZ-ACTIVATED RESISTANCE 1 (*ZAR1*), is required for recognition of the *P. syringae* T3SE HopZ1a. We constructed an *Arabidopsis R* gene T-DNA insertion collection (ARTIC), which was used in a reverse genetic screen to identify *ZAR1*. T-DNA insertions in the *ZAR1* locus result in the loss of HopZ1a recognition, as seen by macroscopic HR assays, trypan blue staining, ion leakage and bacterial growth *in planta*. Using plants mutated in known signaling components *SGT1a*, *SGT1b*, *NDR1*, *RAR1*, *EDS1*, *PAD4*, *RBOHD/F*, *EDS16* or *EDM2*, we demonstrate that HopZ1a-induced immunity employs an uncharacterized ETI signaling pathway. Phylogenetic analyses using the ZAR1 CC domain showed that the closest homologues to ZAR1 are from divergent plant species, including *Ricinus communis* (castor bean), *Populus trichocarpa* (poplar), *Vitis vinifera* (grape) and *Solanum melongen* (eggplant), rather than *Arabidopsis*. Interestingly, in *Arabidopsis* plants genetically lacking *ZAR1*, HopZ1a acts as a virulence factor by promoting bacterial growth, supporting an ancestral virulence function prior to the evolution of ZAR1-mediated immunity. The closely-related HopZ1a family member, HopZ1b, is still recognized in the *zar1* knockout demonstrating that *Arabidopsis* R proteins have diversified to recognize the HopZ family of T3SEs.

## Results

### HopZ1a-induced immunity is independent of known *Arabidopsis* resistance signaling genes

We previously demonstrated that HopZ1a induces a resistance response and an associated hypersensitive response in *Arabidopsis* that is characteristic of effector triggered immunity (ETI) using macroscopic HR assays, trypan blue staining, conductivity assays and bacterial growth assays [35],[39]. Expression of the HopZ1a catalytic mutant (HopZ1a^C216A^, hereafter HopZ1a^C/A^) no longer induced ETI [39]. We further showed that this resistance response is independent of known *R* genes *RPM1*, *RPS2*, *RPS5*, *RPS4*, *RPS6* and the RPM1-interacting protein RIN4 indicating that HopZ1a-induced immunity may involve a novel signaling pathway [39; Lewis *et al.*, unpublished]. To further examine this possibility we investigated HopZ1a-induced immunity in a larger collection of *R* gene-signaling mutant plants (Table 1).

**Table 1:** *Arabidopsis* Resistance signaling genes addressed in this study.

Gene	Ecotype	Function	Reference
*SGT1a*	Ws	R protein accumulation/stability	[26],[32]
*SGT1b*	Col-0	R protein accumulation/stability	[26],[28],[31],[32]
*RAR1*	Col-0	R protein accumulation/stability	[26],[27],[29]
*NDR1*	Col-0	Signaling component of CC type R proteins	[20]–[23]
*EDS1*	Ws	Signaling component of TIR type R proteins	[23],[24]
*PAD4*	Col-0	Interacts with EDS1, accumulation of SA	[25],[63]
*RBOHD/F*	Col-0	Accumulation of reactive oxygen intermediates	[34]
*nahG*	Col-0	Degradation of SA	[33],[40]
*EDS16* (*SID2* or *ICS1*)	Col-0	Plastid-derived SA synthesis	[43]–[45]
*EDM2*	Col-0	Regulates RPP7 expression	[46]
*EDS1* and *SID2* (*EDS1* and *EDS16*)	Col-0/Ws-0		[48]
*NDR1* and *EDS1*	Col-0/Ws-0		[47]

**Figure 1:**
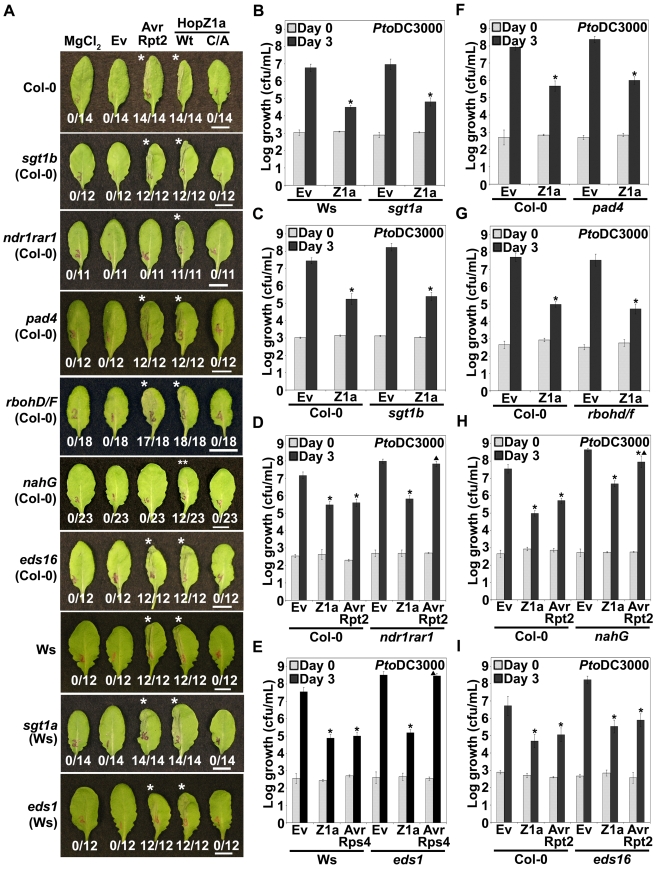
HopZ1a recognition is independent of known signaling components of *R* gene- mediated immunity. (A) Half-leaves of *Arabidopsis* Col-0, Ws-0 or mutant plants were infiltrated with 10 mM MgCl_2_ or with *Pto*DC3000 expressing the empty vector (Ev), AvrRpt2, or HopZ1a or HopZ1a^C216A^ (C/A) with a C-terminal HA tag under its endogenous promoter. C216 of HopZ1a is part of the predicted catalytic triad and the mutant protein is expressed at a similar level to HopZ1a [39]. The bacteria were syringe infiltrated into the leaves at 5×10^7^ cfu/mL. Photos were taken 22 hours post-infiltration. The number of leaves showing an HR is indicated below the appropriate construct. HRs are marked with an asterisk. Patchy HRs are marked with a double asterisk. Scale bar is 1 cm. (B–I) *Pto*DC3000 expressing the indicated construct was syringe infiltrated at 1×10^5^ cfu/mL into *Arabidopsis* Col-0 or mutant leaves and bacterial counts were determined one hour post-infection (Day 0) and 3 days post-infection (Day 3). Two-tailed homoschedastic t-tests were performed to test for significant differences. Within a plant genotype, treatments were compared to empty vector and significant differences are indicated by an asterisk (* P<0.01). To compare between plant genotypes, growth of *Pto*DC3000 carrying HopZ1a, AvrRpt2 or AvrRps4 was normalized to the average growth of *Pto*DC3000(Ev). Significant differences in growth of a *P. syringae* strain between a mutant genotype and wild type Col-0 or Ws are indicated by a triangle (▴ P<0.01). Error bars indicate the standard deviation from the mean of 10 samples. Growth assays were performed at least 3 times. *Arabidopsis* genotypes are: (B) *sgt1a* (C) *sgt1b* (D) *ndr1rar1* (E) *eds1* (F) *pad4* (G) *rbohd/f* (H) *nahG* (I) *eds16*.

We examined the ability of HopZ1a to induce an ETI-associated hypersensitive response (HR) in *Arabidopsis* lines with characterized mutations in various defense signaling and response pathways. We tested *sgt1a*, *sgt1b*, *ndr1rar1*, *eds1* or *pad4* plants by pressure infiltrating each with *P. syringae* pv. tomato DC3000 (*Pto*DC3000) carrying a plasmid encoding *hopZ1a* controlled by its native promoter (Figure 1A). All of these plants displayed a macroscopic HopZ1a-induced HR indicating that these genes do not contribute to HopZ1a-recognition. In contrast, our control infiltration of *Pto*DC3000 carrying the T3SE AvrRpt2 under the *nptII* promoter did not induce an HR in *ndr1rar1* plants as expected [23],[29].

Other genes involved in the defense response against pathogens include *RBOHD* and *RBOHF*, which contribute to reactive oxygen species production [34]. HopZ1a-mediated HR was retained in *rbohd/f* plants. The plant hormone salicylic acid (SA), which plays a number of critical roles in the defense response, is degraded in *nahG* transgenic lines via a bacterial salicylate hydroxylase [40]. The HR induced by HopZ1a was partially compromised in *nahG* plants, with a patchy HR observed in 52% of leaves, whereas the HR induced by AvrRpt2 was completely abrogated [40]. The *nahG* transgene is known to have pleiotropic effects on plant development, and the breakdown products of salicylic acid suppress resistance responses in *Arabidopsis* [41],[42]. We therefore also examined HopZ1a-induced defense responses in *eds16* plants (also called *sid2* or *ics1*) impaired in the isochorismate synthase responsible for the synthesis of SA during plant immunity [43]–[45]. In contrast to *nahG* plants, *eds16* plants still displayed both HopZ1a- and AvrRpt2-mediated HRs. The gene *EDM2* contributes to RPP7-mediated resistance against *H. arabidopsidis* by maintaining transcript levels of *RPP7* [46]. *RPP7* resistance does not depend on salicylic acid [46]. In the *edm2-2* plants, we still observed a HopZ1a-mediated HR (Figure S1).

Some ETI responses have been demonstrated to cooperatively require disease resistance signaling components [47],[48]. We therefore examined several double mutants for the production of the HopZ1a HR. EDS1 and SID2 (or EDS16) are both necessary for resistance mediated by RPS2 against *P. syringae*, RPP8 against *H. arabidopsidis*, and HRT against Turnip Crinkle Virus [48]. *eds1sid2* plants still displayed a HopZ1a-induced HR (Figure S1). We also examined the *ndr1eds1* mutant, which displays slight impairment of RPP7- and RPP8-mediated immunity to *H. arabidopsidis* [47]. We still observed a HopZ1a-induced HR in the *ndr1eds1* mutant (Figure S1). Thus, HopZ1a-induced HR is not dependent on *R* gene-mediated signaling genes *SGT1a*, *SGT1b*, *NDR1*, *RAR1*, *EDS1*, *PAD4*, *RBOHD/F*, *EDS16* or *EDM2*, and does not require the cooperative action of EDS1 and SID2, or EDS1 and NDR1.

To further quantify the extent of HopZ1a-mediated immunity in *Arabidopsis*, we compared the *in planta* growth of the virulent strain *Pto*DC3000 carrying an empty vector (Ev) to the same strain carrying *hopZ1a* under the control of its native promoter over the course of three days. HopZ1a caused a 2.0–3.0 log reduction in growth in *sgt1a*, *sgt1b*, *ndr1rar1*, *eds1* or *pad4* plants comparable to that observed in Ws (for *sgt1a* and *eds1*) or Col-0 (for *sgt1b*, *ndr1rar1* and *pad4*) wild type backgrounds indicating that HopZ1a-mediated resistance is retained in these mutant plants (Figure 1B–1F). As expected, AvrRpt2 and AvrRps4 resistance was abrogated in *ndr1rar1* and *eds1* mutant plants, respectively [23],[29] (Figure 1D and 1E). Similarly, in *rbohD/F* plants *Pto*DC3000(*hopZ1a*) exhibited typical low levels of bacterial growth (4.5–5.0 logs), comparable to the HopZ1a resistance observed in Col-0 wild type plants (Figure 1G). These experiments provide further support that HopZ1a-mediated immunity does not act through *SGT1a*, *SGT1b*, *NDR1*, *RAR1*, *EDS1*, *PAD4*, or *RBOHD/F*.

Consistent with the partial loss of HR observed in *nahG* plants, resistance to both HopZ1a and AvrRpt2 was impaired in the *nahG* transgenic line (Figure 1H). *Pto*DC3000(*hopZ1a*) and *Pto*DC3000(*avrRpt2*) exhibited ∼1.5 log and 2.0–2.5 log more growth in *nahG* than in Col-0 plants, respectively. In contrast to *nahG* plants, *Pto*DC3000(*hopZ1a*) induced a typical defense response in *eds16*, with a 2.0–2.5 log reduction in bacterial growth, similar to wild type Col-0 plants (Figure 1I). *Pto*DC3000(*avrRpt2*) also exhibited a typical defense in *eds16*, as has been previously observed [43]. Our results show that the HopZ1a-induced HR is not dependent on the plastid-source of SA and that partial impairment of resistance in the *nahG* background may be due to the pleiotropic effects of *nahG* on plant development or immunity (see Discussion). In summary, HopZ1a-induced HR and immunity are not dependent on the *R* gene-mediated signaling genes *SGT1a*, *SGT1b*, *NDR1*, *RAR1*, *EDS1*, *PAD4*, *RBOHD/F*, *EDS16* or *EDM2*.

### The type III effector HopZ1a is recognized by the *Arabidopsis* ZAR1 resistance protein

We used a reverse genetics approach to identify the *R* gene responsible for HopZ1a recognition. We generated an *Arabidopsis R* gene T-DNA insertion collection (ARTIC) comprising publicly available T-DNA insertion lines (or if necessary transposon insertion lines) for all of the canonical *R* genes identified from the *Arabidopsis thaliana* Col-0 genome [16],[49]. In order to maximize the chance of obtaining a knock-out line for an individual *R* gene, preference was given to T-DNA or transposon insertion lines with a high confidence insertion in the locus of the gene of interest (and no other known loci) and preferably an insertion in an exon near the beginning of the gene. If there was no T-DNA or transposon insertion in an exon, lines were chosen in the following order of preference: 5′UTR>3′UTR> within 1000 nt upstream of the start codon (1000-promoter)>intron. T-DNA or transposon insertions were available for 166/170 *R* genes. Lines were chosen primarily from the Salk [50] and Sail [51] T-DNA insertion collections, with a few representatives from the WiscDsLox [52], and GT [53] transposon insertion collections. ARTIC includes homozygous individuals from 118 Salk lines, 13 Sail lines and 1 WiscDsLox line, as well as heterozygous individuals from 17 Salk and 7 Sail lines (Table S1).

**Figure 2:**
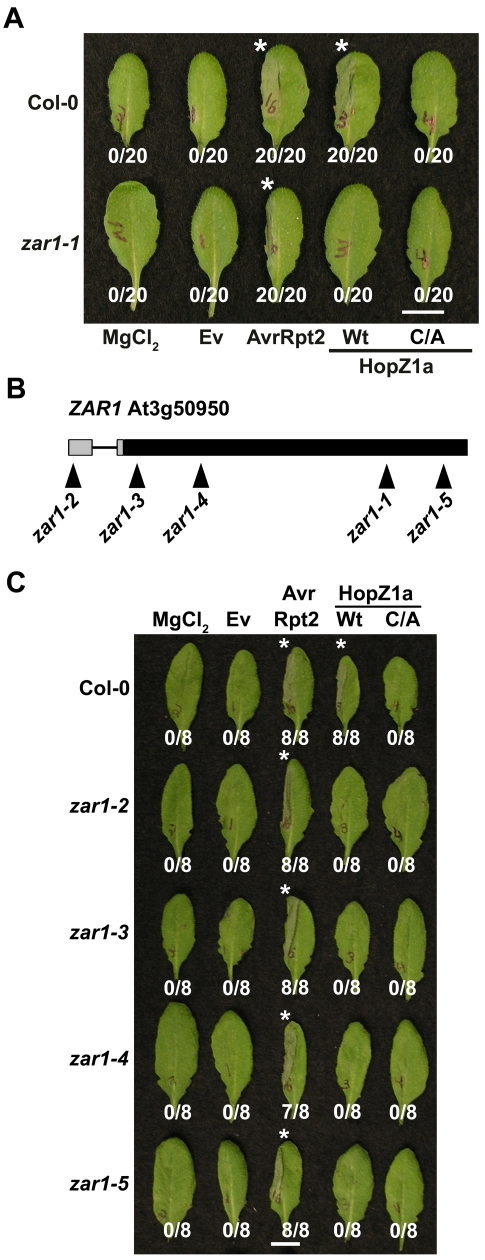
ZAR1 recognizes HopZ1a in *Arabidopsis*. (A) Half-leaves of *Arabidopsis* Col-0 or *zar1-1* plants were infiltrated with 10 mM MgCl_2_ or with *Pto*DC3000 expressing the empty vector (Ev), AvrRpt2, or HopZ1a or HopZ1a^C216A^ (C/A) with a C-terminal HA tag under its endogenous promoter. C216 of HopZ1a is part of the predicted catalytic triad and the mutant protein is expressed at a similar level to HopZ1a [39]. The bacteria were syringe infiltrated into the leaves at 5×10^7^ cfu/mL. Photos were taken 22 hours post-infiltration. The number of leaves showing an HR is indicated below the appropriate construct. HRs are marked with an asterisk. Scale bar is 1 cm. (B) At3g50950 is *ZAR1*. The promoter is shown by grey boxes and the exon by a large black box. There is an intron in the promoter, shown by a black line. The position of the T-DNA insertion lines is shown below the locus. (C) Half-leaves of *Arabidopsis* Col-0, *zar1-2*, *zar1-3*, *zar1-4*, or *zar1-5* plants were infiltrated with 10 mM MgCl_2_ or with *Pto*DC3000 expressing the empty vector (Ev), AvrRpt2, or HopZ1a or HopZ1a^C216A^ (C/A) with a C-terminal HA tag under its endogenous promoter.

To identify the *R* gene responsible for HopZ1a recognition, we infiltrated T-DNA insertion lines from ARTIC with *Pto*DC3000(*hopZ1a*) and screened for a loss of the HopZ1a-induced HR. One line, SALK_013297 (hereafter referred to as *zar1-1*), did not develop a HopZ1a-induced HR but was still competent in initiating an AvrRpt2-mediated HR (Figure 2A). We confirmed by sequencing that the T-DNA insertion in *Arabidopsis zar1-1* plants was found in the gene At3g50950, which we refer to as *HopZ-Activated Resistance* or *ZAR1*. To confirm that ZAR1 was responsible for recognition of HopZ1a, we obtained additional T-DNA insertion lines in At3g50950 and examined them for HopZ1a-induced immunity. We identified four additional alleles of *zar1* (Figure 2B) and genotyped them to identify homozygous lines (data not shown). All of the additional *zar1* T-DNA insertion lines lacked a macroscopic HR in response to *Pto*DC3000(*hopZ1a*) but not *Pto*DC3000(*avrRpt2*) confirming the requirement of ZAR1 for HopZ1a-mediated immunity (Figure 2C). To show that the HopZ1a protein is delivered into *zar1* plant cells, we performed HR assays in Col-0 and *zar1* using a HopZ1a chimeric fusion to the C-terminus of AvrRpt2 (amino acids 80–255) [54], which is recognized by the RPS2 resistance protein in *Arabidopsis* Col-0 [55],[56]. The HopZ1a-AvrRpt2^Δ1-79^ fusion still causes a strong HR in Col-0 and *zar1-1* demonstrating that lack of recognition of HopZ1a in *zar1* plants is not due to lack of HopZ1a translocation (Figure S2). We also tested *zar1* plants for recognition of the endogenous HopZ1a allele carried by *P. syringae* pv. *syringae* strain A2 (*Psy*A2) [35]. In Col-0 plants, *Psy*A2 causes a macroscopic HR (Figure S3) as previously described [35]. In *zar1-1* plants, we no longer observed a macroscopic HR, demonstrating that *zar1* is responsible for recognition of the HopZ1a native strain, *Psy*A2 (Figure S3).

**Figure 3:**
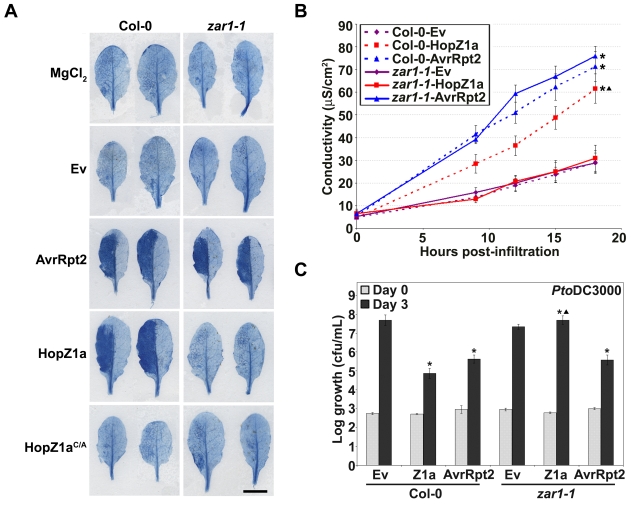
*zar1-1 Arabidopsis* plants do not display immunity against HopZ1a. (A) Trypan blue staining of *Pto*DC3000-infiltrated *Arabidopsis* Col-0 or *zar1-1* leaves. The bacteria were syringe infiltrated into the leaves at 5×10^7^ cfu/mL. Scale bar is 1 cm. C/A indicates the C216A mutation of HopZ1a in the predicted catalytic triad. The mutant protein is expressed at a similar level to HopZ1a [39]. (B) Electrolyte leakage of *Arabidopsis* Col-0 or *zar1-1* leaf discs after infiltration with *Pto*DC3000 expressing the indicated constructs. The bacteria were syringe infiltrated into the leaves at 2×10^7^ cfu/mL. Error bars indicate the standard deviation from the mean of 6 samples. C/A indicates the C216A mutation. Two-tailed homoschedastic t-tests were performed to test for significant differences. Within a plant genotype, treatments were compared to empty vector and significant differences are indicated by an asterisk (* P<0.01). To compare between plant genotypes, ion leakage from *Pto*DC3000 carrying HopZ1a or AvrRpt2 was normalized to the average ion leakage of *Pto*DC3000(Ev) in the same genotype. Significant growth differences between *zar1-1* and wild-type Col-0 are indicated by a triangle (▴ P<0.01). (C) *Pto*DC3000 expressing the indicated construct was syringe infiltrated at 1×10^5^ cfu/mL into *Arabidopsis* Col-0 or *zar1-1* leaves and bacterial counts were determined one hour post-infection (Day 0) and 3 days post-infection (Day 3). Two-tailed homoschedastic t-tests were performed to test for significant differences. Within a plant genotype, treatments were compared to empty vector and significant differences are indicated by an asterisk (* P<0.01). To compare between plant genotypes, growth of *Pto*DC3000 carrying HopZ1a or AvrRpt2 was normalized to the average growth of *Pto*DC3000(Ev). Significant growth differences between *zar1-1* and wild-type Col-0 are indicated by a triangle (▴ P<0.01). Error bars indicate the standard deviation from the mean of 10 samples. Growth assays were performed at least 3 times.

We further verified that HopZ1a-induced immunity and HR were abrogated in *zar1-1* plants via a series of qualitative and quantitative avirulence assays. Trypan blue stain is only retained in dead and/or dying cells, and therefore is a qualitative measure of the HR-associated cell death. Heavy trypan blue staining indicative of an HR was observed in *zar1-1* leaves infiltrated with *Pto*DC3000(*avrRpt2*) and Col-0 leaves infiltrated with *Pto*DC3000(*hopZ1a*) or *Pto*DC3000(*avrRpt2*) at 12 hours post-infection (Figure 3A). However, *zar1-1* leaves infiltrated with *Pto*DC3000(*hopZ1a*) did not result in any significant staining with trypan blue indicating the lack of an HR. As a quantitative measure of the HR, we monitored HR-associated electrolyte leakage as measured by changes in media conductivity (Figure 3B). *Pto*DC3000(*hopZ1a*) or *Pto*DC3000(*avrRpt2*) infiltrated Col-0 leaves increased conductivity by twice as much as *Pto*DC3000(Ev) at 16 hours post-infection, indicative of an HR, and both were significantly different from *Pto*DC3000(Ev) in Col-0 (Figure 3B). In the *zar1-1* mutant, increased conductivity was observed from leaves infiltrated with *Pto*DC3000(*avrRpt2*) but not *Pto*DC3000(*hopZ1a*) (Figure 3B). The conductivity measured from *Pto*DC3000(*hopZ1a*) infiltrated *zar1-1* was significantly different from *Pto*DC3000(*hopZ1a*) in Col-0 and was not significantly different from that of Col-0 or *zar1-1* leaves infiltrated with *Pto*DC3000(Ev), demonstrating that HopZ1a associated electrolyte leakage is abrogated in *zar1* plants.

We monitored HopZ1a-mediated immunity through bacterial growth assays in Col-0 and *zar1-1* plants with *Pto*DC3000 carrying Ev, HopZ1a or AvrRpt2. Bacterial growth was strongly restricted in Col-0 infiltrated with *Pto*DC3000(*hopZ1a*) or *Pto*DC3000(*avrRpt2*) relative to *Pto*DC3000(Ev) (Figure 3C), while HopZ1a-induced immunity was lost in *zar1-1* plants. Importantly, *Pto*DC3000(*hopZ1a*) grew slightly, but significantly, better than *Pto*DC3000(Ev) in *zar1-1* plants indicative of a virulence function for HopZ1a in *Arabidopsis* plants lacking *ZAR1*. Loss of immunity in *zar1-1* plants was specific to HopZ1a as AvrRpt2 still caused a strong restriction of bacterial growth in *zar1-1* plants similar to that observed in Col-0 plants.

Taken together, our data demonstrates that the ZAR1 R protein specifically recognizes HopZ1a in *Arabidopsis* since it is required for the macroscopic HR, rapid ion leakage, and restricted bacterial proliferation induced by HopZ1a. Further, ZAR1 is necessary for recognition of HopZ1a from its native *P. syringae* strain, *Psy*A2.

### HopZ1a has a virulence function in *zar1 Arabidopsis* plants

**Figure 4:**
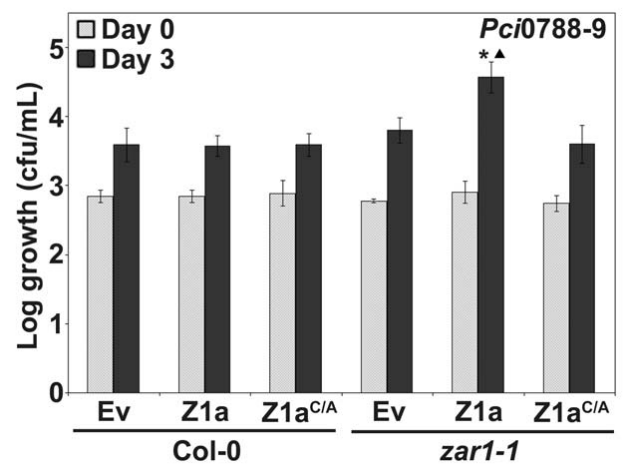
HopZ1a has a virulence function in *zar1-1 Arabidopsis* plants. *Pci*0788-9 expressing the indicated construct was syringe infiltrated at 1×10^5^ cfu/mL into *Arabidopsis* Col-0 or *zar1-1* leaves and bacterial counts were determined one hour post-infection (Day 0) and 3 days post-infection (Day 3). C/A indicates the C216A mutation of HopZ1a in the predicted catalytic triad and the mutant protein is expressed at a similar level to HopZ1a [39]. Two-tailed homoschedastic t-tests were performed to test for significant differences. Within a plant genotype, treatments were compared to empty vector and significant differences are indicated by an asterisk (* P<0.01). To compare between plant genotypes, growth of *Pci*0788-9 carrying HopZ1a, or HopZ1a^C216A^ (HopZ1a^C/A^) was normalized to the average growth of *Pci*0788-9(Ev). Significant differences between *zar1-1* and Col-0 are indicated by a triangle (▴ P<0.01). Error bars indicate the standard deviation from the mean of 10 samples. Growth assays were performed at least 3 times.

The observation that *Pto*DC3000(*hopZ1a*) displayed slightly enhanced growth in *zar1-1* relative to *Pto*DC3000(Ev) prompted us to further investigate whether HopZ1a displays a virulence function in *Arabidopsis* plants lacking ZAR1 (Figure 3C). We used the non-host strain *P. syringae* pv. cilantro 0788-9 (hereafter *Pci*0788-9) as it does not carry an endogenous HopZ allele and is closely-related to *P. syringae* pv. *maculicola* ES4326 which carries a HopZ1c allele [35]. Further, we previously demonstrated that the related HopZ2 effector displays an enhanced virulence function in *Arabidopsis* ecotype Col-0 when delivered by *Pci*0788-9 [39]. We infiltrated *Pci*0788-9 carrying HopZ1a, HopZ1a^C/A^, or empty vector into *zar1-1* and Col-0 plants and determined the level of bacterial proliferation after three days of growth. *Pci*0788-9(*hopZ1a*) exhibits a significant 0.5–0.75 log increase in growth compared to *Pci*0788-9(Ev) in *zar1-1* (Figure 4). Since the catalytic cysteine residue of HopZ1a was previously shown to be necessary for *R* gene-mediated recognition (Figure 2A) and enzymatic activity [35],[39], we investigated whether enzymatic activity of HopZ1a is also necessary for virulence, and showed that the catalytic mutant *Pci*0788-9(*hopZ1a^C/A^*) grows to the same level as the vector control *Pci*0788-9(Ev) (Figure 4). We also confirmed that ZAR1-mediated resistance in Col-0 was not observed with the weakly virulent *Pci*0788-9, by showing that *Pci*0788-9(Ev), *Pci*0788-9(*hopZ1a*) and *Pci*0788-9(*hopZ1a^C/A^*) grew to equivalent low titers after three days [37]. Thus, HopZ1a promotes bacterial proliferation in the absence of ZAR1 recognition.

### The ZAR1 coiled-coil domain is widespread, yet evolutionarily distinct from other R proteins

ZAR1 is a CC-NBS-LRR type R protein that has an evolutionary history unique from other *R* genes in the Col-0 genome. Previous phylogenetic analysis using the NBS domain of *Arabidopsis* R proteins indicated that the most similar resistance proteins to ZAR1 are homologues of RPP13, a downy mildew resistance protein originally identified in the Niederzenz (Nd-1) ecotype of *Arabidopsis* [16],[57]. While ZAR1 and RPP13 are both clustered into the CNL-C subgroup of NBS-containing proteins their divergence is quite ancient, and in fact ZAR1 shares the same common ancestor with RPP13 as it does with RPP8. Despite this, Meyers et al. [16] classified *RPP8* and related sequences as a different subgroup (CNL-D) since they have two introns, while *ZAR1*, *RPP13*, and *RPP13*-related sequences have no introns.

**Figure 5:**
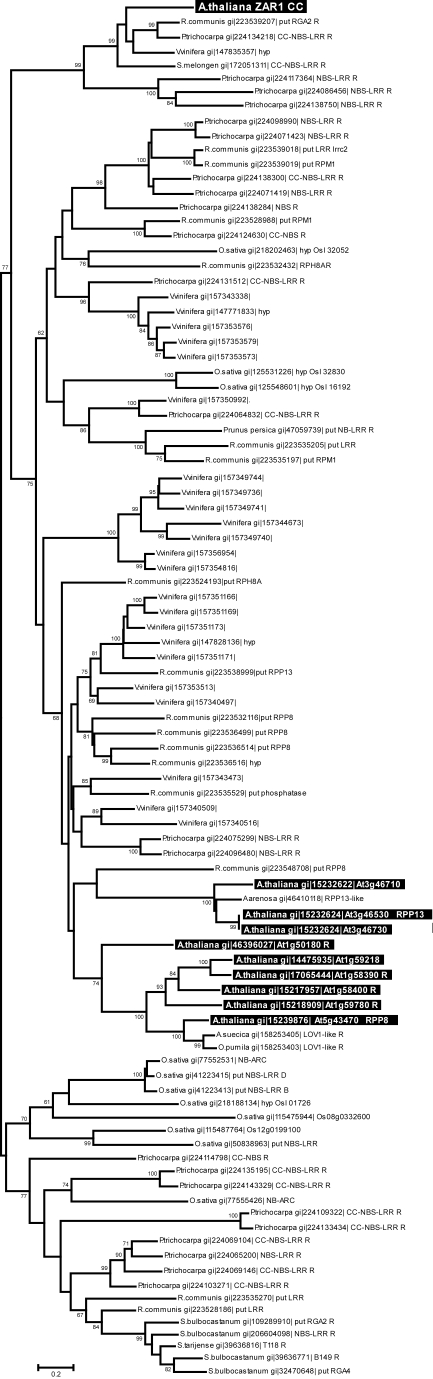
Evolutionary relationships of 95 ZAR1 coiled-coil domain homologs. The evolutionary relationships of the homologous amino acid sequences were inferred using Neighbor-Joining, with the robustness of the tree assessed via bootstrapping (500 replicates, with bootstrap values greater than 60% shown above the appropriate nodes). The tree is drawn to scale, with branch lengths scaled to evolutionary distances (scale shown at the bottom of the tree). All *Arabidopsis* ZAR1 coiled-coil domain homologs are shown in reverse type, while the ZAR1 sequence is found at the top of the tree. The data were parsed to remove redundant sequences as described in the Materials and Methods. “put” indicates a putative R protein while “hyp” is hypothetical. The major structure of this tree (e.g. clustering of ZAR1 and other *Arabidopsis* homologs) is identical to that observed in trees produced by maximum likelihood and maximum parsimony analysis (data not shown).

Since the R proteins RPM1, PRF and RPS5 interact through their N-terminal coiled-coil (CC) domain with the T3SE-targeted host protein which they monitor [58]–[60], we reanalyzed the phylogenetic relationships among plant R proteins using only the CC domains, which may provide a basis for identifying *R* genes that could monitor similar protein families (Figure 5). Bootstrapped neighbor-joining, maximum likelihood, and maximum parsimony analyses provided highly congruent results that identified closely-related *ZAR1* homologs in four species, *Ricinus communis* (castor bean), *Populus trichocarpa* (poplar), *Vitis vinifera* (grape), and *Solanum melongen* (eggplant) (Figure 5, Figure S4). The other *Arabidopsis* R proteins are highly divergent in their CC domains from ZAR1, and are found in a large distinct and well-supported clade that includes the highly diverse *Arabidopsis RPP13* and *RPP8* protein families as well as homologues from several other species (Figure 5). While the lack of a reliable root for the phylogenetic analysis complicates the interpretation of the tree, it is clear that the ZAR1 clade is significantly distinct (as shown by bootstrap analysis) from the rest of the CC domain tree.

### ZAR1 does not recognize the very closely related HopZ1b allele

**Figure 6:**
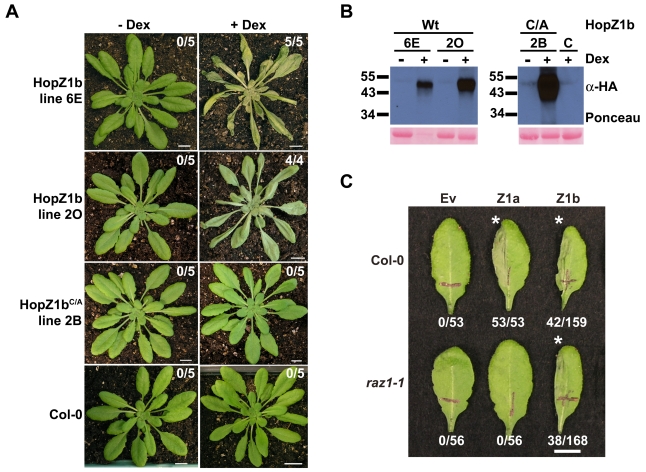
ZAR1 does not recognize HopZ1b. (A) Transgenic homozygous HopZ1b or HopZ1b^C/A^ plants were sprayed with 30 µM dexamethasone or water. C/A indicates the C212A mutation of HopZ1b in the predicted catalytic triad. Photos were taken 24–72 hours post-spraying. The number of plants showing a macroscopic HR is indicated in each box. Scale bar is 1 cm. (B) Immunoblot analysis of HopZ1b or HopZ1b^C/A^ protein expressed in transgenic lines after treatment with 30 µM dexamethasone or water. C/A indicates the C212A mutation of HopZ1b in the predicted catalytic triad. The Ponceau Red stained blot serves as the loading control. The predicted size of HopZ1b-HA is 42.4 kDa. (C) Half-leaves of *Arabidopsis* Col-0 or *zar1-1* plants were infiltrated with 10 mM MgCl_2_ or with *Pto*DC3000 expressing the empty vector (Ev), HopZ1a or HopZ1b with a C-terminal HA tag under its endogenous promoter. The bacteria were syringe infiltrated into the leaves at 5×10^7^ cfu/mL. Photos were taken 24 hours post-infiltration. The number of leaves showing an HR is indicated below the appropriate construct. HRs are marked with an asterisk. Scale bar is 1 cm.

We previously demonstrated that HopZ1b induces an HR in ∼24% of *Arabidopsis* Col-0 leaves when delivered by *Pto*DC3000 [39]. To further demonstrate that HopZ1b causes an HR, we generated dexamethasone-inducible transgenic HopZ1b plants and tested these for production of the HR upon HopZ1b expression. Two independent HopZ1b transgenic lines induced a strong whole-plant HR within 24–48 hours of dexamethasone-application (Figure 6A). We also tested a HopZ1b^C212A^ transgenic line for production of the HR. HopZ1b^C212A^ did not induce an HR, indicating that the enzymatic activity is necessary for HopZ1b recognition (Figure 6A). The HopZ1b and HopZ1b^C212A^ proteins were all detectable only after application of dexamethasone (Figure 6B).

Given that HopZ1a and HopZ1b are 75% identical at the nucleotide level and 72% identical at the amino acid level, we investigated whether ZAR1 also recognized HopZ1b. We infiltrated Col-0 or *zar1-1* with *Pto*DC3000 carrying Ev, HopZ1a or HopZ1b and monitored for the development of an HR. As expected, 100% of leaves infiltrated with *Pto*DC3000(*hopZ1a*) developed an HR in Col-0 and no leaves developed an HR in *zar1-1*; however, when infiltrated with *Pto*DC3000(*hopZ1b*), only 26% of Col-0 leaves and 23% of *zar1-1* leaves developed an HR (Figure 6C). HopZ1b therefore causes a macroscopic HR in *Arabidopsis* Col-0, which like HopZ1a is dependent on its enzymatic activity. However, HopZ1b recognition is not mediated by ZAR1 and must be conferred by a distinct *R* gene.

## Discussion

Resistance proteins are an integral and essential component of the plant immune system. They provide a flexible and readily adaptable means for plants to recognize pathogens that are able to suppress or bypass basal immune responses. In *Arabidopsis thaliana* alone, there are ∼170 *R* genes; however, resistance specificities have been determined for relatively few (Table S1). The *Arabidopsis R* gene T-DNA Insertion Collection (ARTIC) provides a resource to rapidly query the Arabidopsis resistance genome for particular *R* gene functions. In support of this, we used ARTIC in a reverse genetic screen to identify the CC-NB-LRR resistance protein ZAR1, required for recognition of the *P. syringae* T3SE HopZ1a.

*R* genes are frequently present in diverse clusters within a genome [16], which may allow them to evolve new specificities against pathogens through recombination, gene conversion, or by other mutational mechanisms [14] in response to the selection pressure imposed during the infection process [61]. It is also common to find very high diversity in *R* genes due to pathogen-driven selective diversification. However ZAR1 is not part of a genomic cluster of similar *R* genes, and unlike the closely-related RPP13 and RPP8 families, no highly similar homologs of the CC domain are found in the *Arabidopsis* genome (Figure 5, Figure S4). The data available to date indicates that within the ZAR1 CC domain clade, the only homolog to have undergone extensive diversification is found in *P. trichocarpa*. None of the other species in the ZAR1 CC domain clade carry more than a single homolog, which is again unusual for this family of proteins. This raises the very intriguing possibility that extensive genetic diversity was not selected for in the ancestral ZAR1 CC domain. High genetic diversity, both with respect to gene family expansion as well as maintenance of allelic diversity, is very commonly observed in genes associated with pathogen recognition and immune response. Given the relative paucity of diversity within the ZAR1 CC domain clade, it is possible that this protein or domain was only relatively recently recruited by the plant immune system, perhaps as a means to track HopZ family diversification. This is not to say that the ZAR1 protein has a recent origin, only that it may have originally served an alternative function not directly associated with ETI. What makes this speculation particularly intriguing is that it is at odds with the observation of Ma *et al.* [35] who showed that HopZ1a is most similar to the ancestral allele of the *P syringae* HopZ family. It will therefore be interesting to determine if ZAR1 homologs from the other species within the ZAR1 CC domain clade also recognize HopZ1a in these diverse hosts, or if recognition is due to other R proteins.

The majority of R proteins characterized to date require NDR1, EDS1, or PAD4 for proper defense induction. ZAR1 is a notable exception to this rule, along with its relatives which recognize isolates of *H. arabidopsidis*, *RPP13* from the Niederzenz (Nd) ecotype [57], *RPP8* from ecotype Landsberg *erecta*, and the *RPP7 R* gene from ecotype Col-0 [23],[47]. For example, the Emco5 isolate of *H. arabidopsidis* induces typical levels of resistance when tested in *Arabidopsis ndr1*, *pad4* or *eds1* mutants transformed with the *RPP13* Nd allele [57], and in *ndr1* or *eds1* mutants transformed with the *RPP8* Ler allele [47].

Does the lack of NDR1, EDS1, and PAD4 dependence in ZAR1, RPP8, or RPP13 indicate that they signal through the same pathway? Further analysis of these R proteins has demonstrated functional redundancy which may help to answer this question. For example, while RPP8- or RPP7- mediated immunity against *H. arabidopsidis* is not impaired in single *ndr1* or *eds1* mutant backgrounds, resistance decreases in the *ndr1eds1* double mutant [47]. Similarly, RPP8-, HRT-, and RPS2-mediated immunity require both EDS1 and SA, as resistance is lost in *eds1nahG* or *eds1sid2* mutants (*sid2* is also known as *eds16*) [48]. Importantly, ZAR1-mediated immunity differs from RPP8, RPP7, HRT, or RPS2 in that immunity is not impaired in *eds1sid2* or *ndr1eds1* double mutants (Figure S1). Additionally, unlike ZAR1, HRT requires PAD4 and EDS1 [62], RPS2 depends on NDR1 [23] and RPP7 requires EDM2 [46]. Several R proteins against *H. arabidopsidis* (RPP2A/B, RPP4, RPP5, RPP7, RPP8) are known to act through SGT1 and/or RAR1 [29],[31]. In contrast, we did not observe any impairment in ZAR1-mediated plant immunity in *sgt1a*, *sgt1b* or *rar1* mutants (Figure 1). These differences in genetic requirements for ZAR1-mediated immunity suggest that its signaling network is quite different from the characterized networks of other R proteins. Interestingly, the only R protein that also acts independently of the known defense signaling pathways is the closely-related RPP13. At this point we do not know if ZAR1 and RPP13 signal through a common pathway.

We also observed a partial impairment of HopZ1a-induced resistance and a complete loss of AvrRpt2-induced resistance in the *nahG* background (Figure 1A and 1H). However, *nahG* has been reported to affect non-host resistance in *Arabidopsis* to *P. syringae* pv. phaseolicola, due to the accumulation of catechol [42]. As well, the *nahG* transgene impairs ethylene signaling, early induction of jasmonate signaling and camalexin production [41]. We therefore tested additional mutants in the SA signaling pathway to clarify these results. The *eds16* mutant, which lacks plastid-derived SA [45], did not impair HopZ1a- or AvrRpt2-mediated resistance responses (Figure 1A and 1I). The *pad4* mutant, which is impaired in SA signaling [63] and has reduced camalexin and ethylene levels [41], exhibits normal HopZ1a-induced resistance (Figure 1A and 1F). We therefore conclude that SA is not involved in HopZ1a-mediated resistance, and that the impairment in the *nahG* background is likely due to the accumulation of catechol or the pleiotropic effects of the *nahG* transgene.

The closely-related HopZ1b allele is only recognized in ∼24% of *Arabidopsis* ecotype Col-0 leaves in contrast to 100% recognition of HopZ1a (Figure 6C). HopZ1b causes a strong HR when overexpressed in transgenic plants and the HR is dependent on the catalytic cysteine (Figure 6A and 6B). Our data strongly support that HopZ1b is recognized by a distinct *R* gene. Thus, recognition specificity for the two HopZ1 alleles may have evolved independently. Our phylogenetic analysis provides strong *R* gene candidates to assay for recognition of HopZ1a in diverse hosts, as well as HopZ1b recognition in *Arabidopsis*.

HopZ1a demonstrates a virulence function in the *zar1* Col-0 background that is dependent on its catalytic function (Figure 4). This virulence function is the putative ancestral state, prior to the development of resistance by the plant. In support of this, recognition of HopZ1a is dependent on its predicted catalytic residues, indicating that HopZ1a is indirectly recognized by ZAR1 via its enzymatic activity. It remains to be determined whether HopZ1a virulence and avirulence activities converge on common or distinct host targets. We previously showed that HopZ2 also has a virulence function in *Arabidopsis* [39], although it is not clear if HopZ1a and HopZ2 target the same host protein to promote bacterial fitness. HopZ1a and HopZ2 have quite different evolutionary histories; HopZ1a, HopZ1b and HopZ1c evolved by pathoadaptation in response to the host immune system, while HopZ2 was acquired by horizontal gene transfer and is most similar to homologues in *Xanthomonas* spp., including AvrRxv [35],[64],[65]. Comparing the host targets of HopZ1a and HopZ2 will allow us to evaluate the extent of diversification of HopZ virulence strategies in *Arabidopsis*.

## Materials and Methods

### Plant materials and growth conditions

*Arabidopsis thaliana* plants were grown with 9 h of light (∼130 microeinsteins m^−2^ s^−1^) and 15 h of darkness at 22°C in Promix soil supplemented with 20∶20∶20 fertilizer. Unless otherwise indicated, assays were performed in the Col-0 background. T-DNA insertion lines were identified using SIGnAL (Salk Institute Genomic Analysis Laboratory) and obtained from the ABRC (*Arabidopsis* Biological Resource Center). All generated homozygous lines have been deposited at the ABRC.

For the *ZAR1* alleles, *zar1-1* is SALK_013297, *zar1-2* is SALK_091754, *zar1-3* is SALK_033548, *zar1-4* is SALK_046916 and *zar1-5* is SALK_009040. The following mutants were utilized: *sgt1a* (in Ws) [32], *sgt1b* (in Col-0) [28], *ndr1-1 rar1-21* (in Col-0) [20],[27], *eds1-1* (in Ws) [66], *pad4-1* (in Col-0) [67], *rbohD/F* (in Col-0) [34], *eds16* (in Col-0) [43],[44], *edm2-2* (in Col-0) [46], *eds1-1sid2-1* (Col-0/Ws-0 cross) [48], *ndr1-1eds1-2* (Col-0/Ws-0 cross) [47], and the transgenic line *nahG* (in Col-0) [40].

### Genotyping of T–DNA insertion lines

Primers were designed using the iSct feature in the SIGnAL database. Primer sequences are available upon request. PCR-based genotyping was employed to determine the homozygosity or heterozygosity of the individuals. Genomic DNA was extracted from a leaf of 5–6 week old *Arabidopsis* plants and PCR products were sequenced using Big Dye Terminator 3.1 on an ABI 3730 genetic analyzer.

### *P. syringae* infection assays

The HopZ1a allele was amplified from the *Pseudomonas syringae* pv. syringae A2, expressed under its native promoter and contained an in-frame hemagglutinin (HA) tag at the C-terminus [39]. *Pseudomonas syringae* pv. tomato DC3000 (*Pto*DC3000) or *Pseudomonas syringae* pv. cilantro 0877-9 (*Pci*0788-9) carried empty vector (pUCP20) [39], pDSK519-P*_nptII_*:*AvrRpt2* [68], pUCP20-P*_hopZ1a_*::*hopZ1a*-HA, pUCP20-P*_hopZ1a_*::*hopZ1a^C216A^*-HA or pUCP20-P*_hopZ1b_*::*hopZ1b*-HA [39] or pV316-1a (carries AvrRps4) [69]. *P. syringae* pv. syringae A2 contains the endogenous HopZ1a allele [35],[39]. HR, ion leakage and in planta growth assays were performed as has been described [39]. For infiltrations, *P. syringae* was resuspended to an OD_600_ = 0.1 (∼5×10^7^ cfu/mL) for HR assays and trypan blue staining, or diluted to 2×10^7^ cfu/mL for ion leakage assays, or diluted to 1×10^5^ cfu/mL for growth curves. Diluted inocula were hand-infiltrated using a needleless syringe as has been described [70]. The HR was scored at 16–20 hours. Leaves for trypan blue staining were harvested at 17–18 hours [39]. For ion leakage assays, 4 disks (1.5 cm^2^) were harvested, soaked in dH_2_0 for 45 minutes and transferred to 6 mL of dH_2_0. Readings were taken with an Orion 3 Star conductivity meter (Thermo Electron Corporation, Beverly, MA). For growth assays, 4 disks (1 cm^2^) were harvested, ground in 10 mM MgCl_2_, and plated on KB with rifampicin and cyclohexamide on day 0 and day 3 for colony counts.

Two-tailed homoschedastic t-tests were performed within genotypes to detect statistical significance. To compare between genotypes, log growth or conductivity was normalized to the average growth or conductivity of *Pto*DC3000(Ev) or *Pci*0788-9(Ev) in the appropriate genotype and two-tailed homoschedastic t-tests were performed.

### Cloning

The HopZ1a-AvrRpt2 fusion was constructed using a crossover PCR approach, as previously described [39],[71]. For the promoter-full length HopZ1a-HA-AvrRpt2^Δ1-79^ fusions, the 5′ portion of the fusion was amplified by PCR using a 5′ primer to the HopZ1a promoter and a 3′ primer to the HA tag, plus a portion of the 5′ end of the AvrRpt2 truncation (Δ1-79) [54]. The 3′ portion of the fusion was amplified by PCR using a 5′ primer to the AvrRpt2 truncation plus a portion of 3′ end of the HA tag, and a 3′ primer to AvrRpt2. These two PCR products were then mixed to use as template for the subsequent PCR reaction. The full-length promoter-HopZ1a-HA-AvrRpt2^Δ1-79^ cassette was amplified using the same 5′ promoter primer and 3′ AvrRpt2 primer and blunt-end cloned into pUCP20 into the *Sma*I site [39]. The promoter-ATG-AvrRpt2^Δ1-79^ fusion, driven by the HopZ1a promoter but lacking the signal and translocation sequence, was previously described [39].

To clone into the pBD vector, HopZ1b or HopZ1b^C212A^ with an in-frame HA tag was amplified by PCR using primers to add a unique *Xho*I site to the 5′ end of the gene and a unique *Spe*I site to the 3′ end of the HA tag [39]. The pBD vector (a gift from Dr. Jeff Dangl, University of North Carolina, Chapel Hill, NC, USA) was modified from pTA7002 to add an HA tag in the multi-cloning site as has been described [58],[72].

### Phylogenetic analysis

ZAR1 homologs were identified from the NCBI nr database via BLASTP analysis using the *Arabidopsis* ZAR1 protein sequence as the query and default parameters. All similar, full-length sequences with an Expect-value below 10^−5^ were downloaded. Full length protein sequences were aligned via MAFFT [73] using the E-INS-i algorithm. Coiled-coil domains were then manually examined and extracted from the sequence using GeneDoc, and the alignment was repeated using the MAFFT G-INS-I algorithm. Following alignment, redundant sequences were removed from the dataset via a custom PERL script (written by DSG). Redundant sequences were defined as those sequences from the same species that have more than 95% amino acid identity. The exception to this was *A. thaliana*, where all Col-0 homologs were retained for the analysis. Neighbor-joining and maximum parsimony phylogenetic analyses were performed with MEGA4 [74] with bootstrapping (1000 pseudo-replicates) and the JTT substitution model. All positions containing alignment gaps were eliminated on a pairwise basis, with a total of 217 positions used in the final dataset. The tree was rooted at the midpoint. Maximum likelihood analysis was performed using the PALM (Phylogenetic Reconstruction by Automated Likelihood Model Selector) [75] server, which performs automated evolution model selection via ProTest [76], and maximum likelihood analysis via PhyML [77]. The best model was determined by AIC to be JTT+G+F.

### Transgenic lines

Col-0 plants were transformed with pBD::*hopZ1b*-HA or pBD::*hopZ1b(C212A)*-HA using the floral dip method [78]. Transgenic plants were selected by Basta resistance and confirmed by PCR and sequencing to have the correct transgene. Homozygosity of T3 lines was determined by their segregation ratios on plates containing half-strength Murashige and Skoog (MS) media and 6 mg/L bialophos. For the Westerns, leaves were detached from the plants and floated on 30µM dexamethasone or water for 48 hours, and frozen in liquid nitrogen. The leaf tissue was ground in a buffer containing 20mM Tris (pH 8.0), 100mM NaCl, 1mM DTT and 1% Triton X-100. The crude extract was cleared by centrifugation at 5000g for 10 minutes at 4°C. After adding SDS-PAGE loading dye and boiling for 5 minutes, 7.5 µL of protein was separated on 12% SDS-PAGE gels, blotted onto nitrocellulose membranes and detected using HA antibodies (Roche) by chemiluminescence (Amersham Biosciences). Photographs were taken 24–72 hours after spraying 30µM dexamethasone (Sigma) or water onto the plants.

## Supporting Information

Figure S1 HopZ1a recognition is independent of known signaling components of *R* gene- mediated immunity. Half-leaves of *Arabidopsis* mutant plants were infiltrated with 10 mM MgCl_2_ or with *Pto*DC3000 expressing the empty vector (Ev), or HopZ1a or HopZ1a^C216A^ (C/A) with a C-terminal HA tag under its endogenous promoter. C216 of HopZ1a is part of the predicted catalytic triad and the mutant protein is expressed at a similar level to HopZ1a [39]. The bacteria were syringe infiltrated into the leaves at 5×10^7^ cfu/mL. Photos were taken 22 hours post-infiltration. The number of leaves showing an HR is indicated below the appropriate construct. HRs are marked with an asterisk. Scale bar is 1 cm.
(3.88 MB TIF)

Figure S2 HopZ1a is translocated into *zar1* plants. Half-leaves of *Arabidopsis* Col-0 or *zar1-1* plants were infiltrated with 10 mM MgCl_2_ or with *Pto*DC3000 expressing the empty vector (Ev), HopZ1a, AvrRpt2, HopZ1a-AvrRpt2^Δ1-79^, or AvrRpt2^Δ1-79^. Full-length AvrRpt2 is driven by the nptII promoter. HopZ1a-AvrRpt2^Δ1-79^ is an in-frame fusion to the HA tag followed by the C-terminus of AvrRpt2 under the HopZ1a promoter. AvrRpt2^Δ1-79^ with an N-terminal in-frame start codon is driven by the HopZ1a promoter. P indicates the promoter. The bacteria were syringe infiltrated into leaves at 5×10^7^ cfu/mL. Photos were taken 22 hours post-infiltration. The number of leaves showing an HR is indicated below the appropriate construct. HRs are marked with an asterisk. Scale bar is 1 cm.
(2.24 MB TIF)

Figure S3 *P. syringae* pv. syringae strain A2 is not recognized in *zar1* plants. Half-leaves of *Arabidopsis* Col-0 or *zar1-1* plants were infiltrated with 10 mM MgCl_2_ or with *Pto*DC3000 expressing HopZ1a (*Pto*+HopZ1a) or *Psy*A2 which endogenously possesses the HopZ1a allele. The bacteria were syringe infiltrated into the leaves at 5×10^7^ cfu/mL. Photos were taken 22 hours post-infiltration. The number of leaves showing an HR is indicated below the appropriate construct. HRs are marked with an asterisk. Scale bar is 1 cm.
(2.57 MB TIF)

Figure S4 Maximum likelihood phylogenetic analysis of the coiled-coil domain from the ZAR1 protein. The tree was constructed based on a MAFFT alignment (E-INS-i algorithm) using the PALM server [75]. The best amino acid substitution model was identified by AIC criterion to be JTT+G+F, with alpha = 2.64. The initial tree was constructed using neighbor-joining, and the final tree was bootstrapped 500 times. All bootstrap scores >50 are presented above the appropriate nodes.
(1.55 MB TIF)

Table S1 *Arabidopsis R* Gene T–DNA Insertion Collection (ARTIC).
(0.52 MB DOC)
