# Supplementary figures and images for: Allele-Specific Virulence Attenuation of the Pseudomonas syringae HopZ1a Type III Effector via the Arabidopsis ZAR1 Resistance Protein
Source: PLoS Genet. 2010 Apr 1;6(4):e1000894. doi: 10.1371/journal.pgen.1000894 (PMC2848558; doi:10.1371/journal.pgen.1000894)

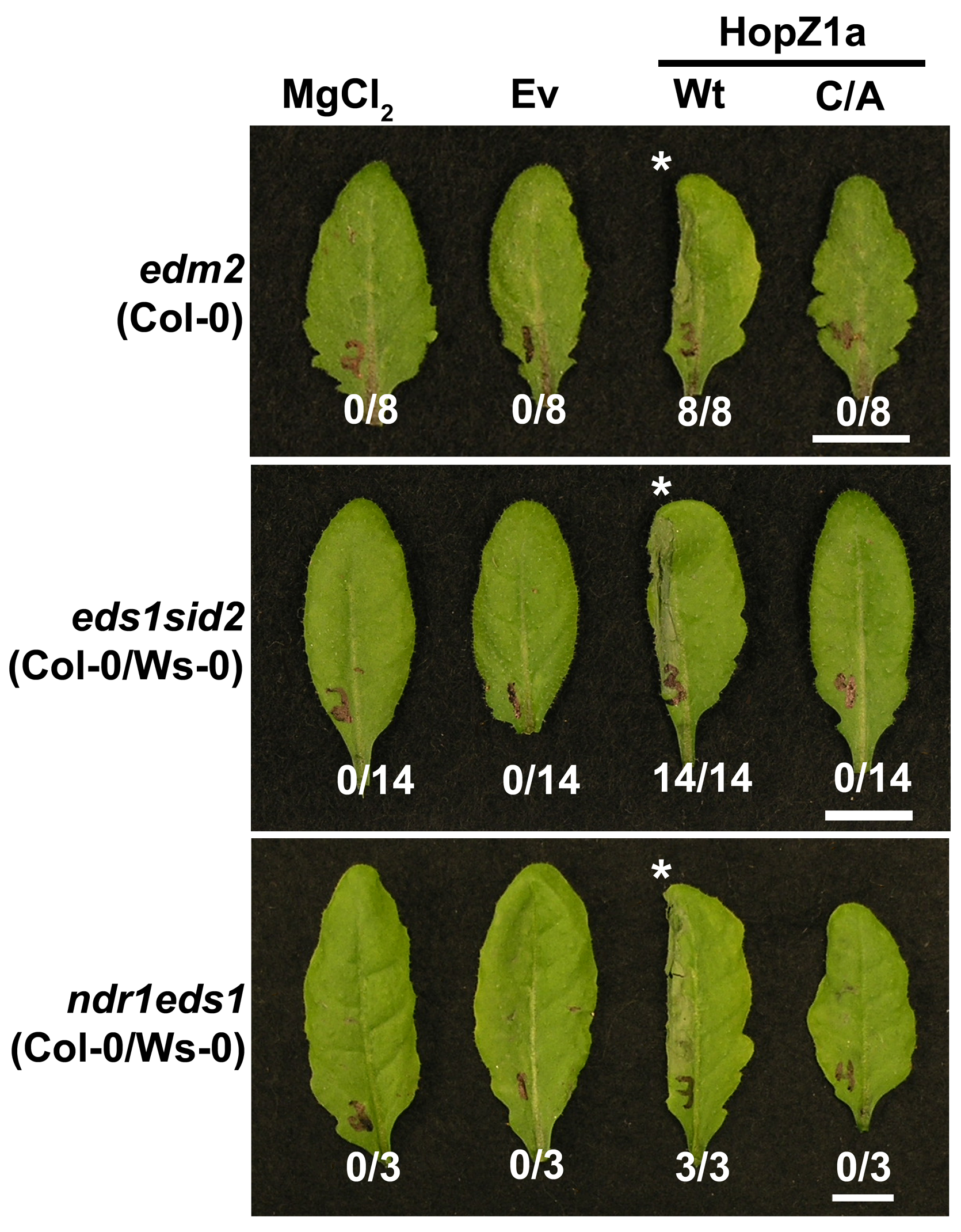

Supplement: Figure S1 — HopZ1a recognition is independent of known signaling components of R gene- mediated immunity. Half-leaves of Arabidopsis mutant plants were infiltrated with 10 mM MgCl2 or with PtoDC3000 expressing the empty vector (Ev), or HopZ1a or HopZ1aC216A (C/A) with a C-terminal HA tag under its endogenous promoter. C216 of HopZ1a is part of the predicted catalytic triad and the mutant protein is expressed at a similar level to HopZ1a [39]. The bacteria were syringe infiltrated into the leaves at 5×10^7^ cfu/mL. Photos were taken 22 hours post-infiltration. The number of leaves showing an HR is indicated below the appropriate construct. HRs are marked with an asterisk. Scale bar is 1 cm. (3.88 MB TIF) [file pgen.1000894.s001.tif]

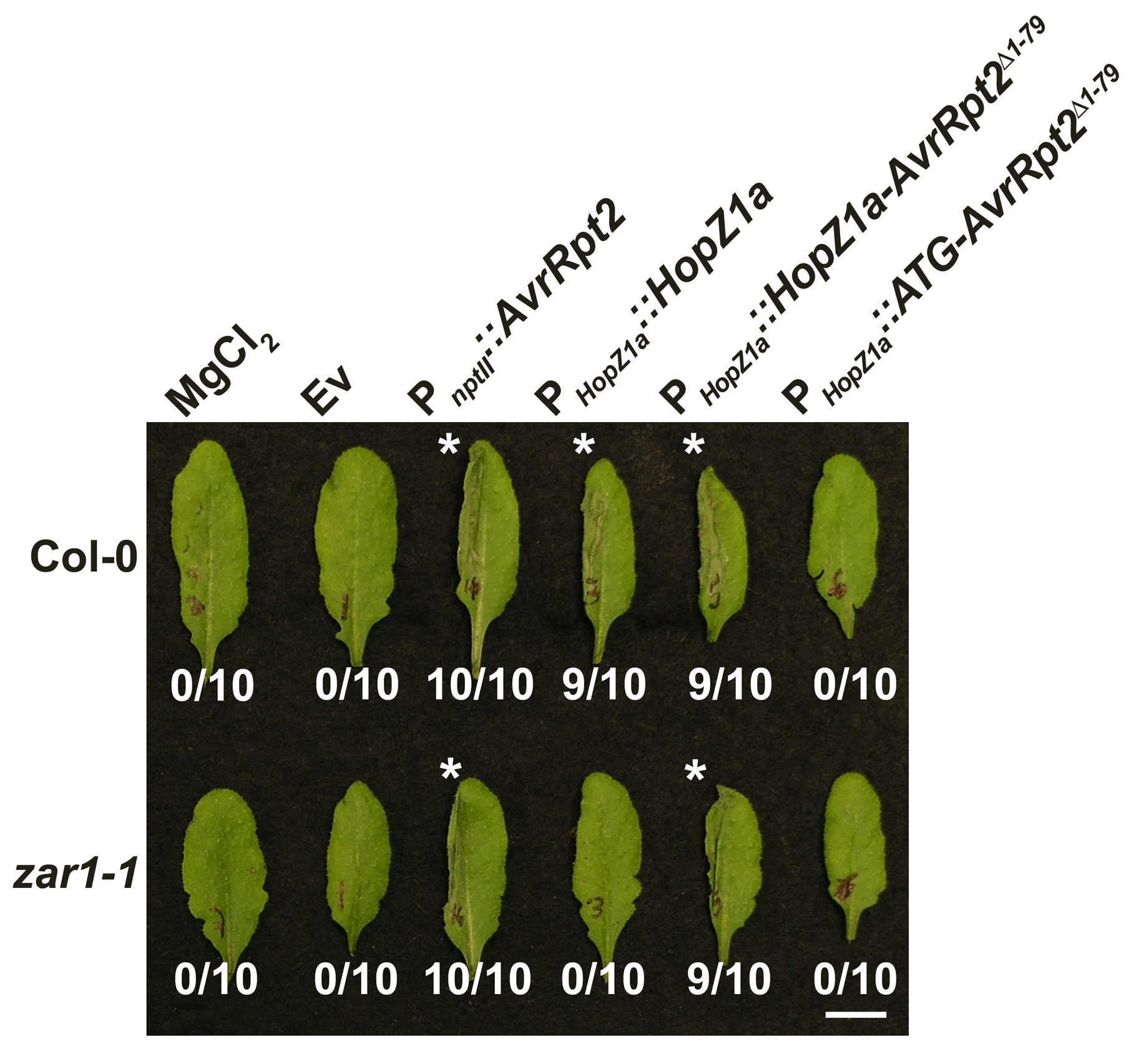

Supplement: Figure S2 — HopZ1a is translocated into zar1 plants. Half-leaves of Arabidopsis Col-0 or zar1-1 plants were infiltrated with 10 mM MgCl2 or with PtoDC3000 expressing the empty vector (Ev), HopZ1a, AvrRpt2, HopZ1a-AvrRpt2Δ1-79, or AvrRpt2Δ1-79. Full-length AvrRpt2 is driven by the nptII promoter. HopZ1a-AvrRpt2Δ1-79 is an in-frame fusion to the HA tag followed by the C-terminus of AvrRpt2 under the HopZ1a promoter. AvrRpt2Δ1-79 with an N-terminal in-frame start codon is driven by the HopZ1a promoter. P indicates the promoter. The bacteria were syringe infiltrated into leaves at 5×10^7^ cfu/mL. Photos were taken 22 hours post-infiltration. The number of leaves showing an HR is indicated below the appropriate construct. HRs are marked with an asterisk. Scale bar is 1 cm. (2.24 MB TIF) [file pgen.1000894.s002.tif]

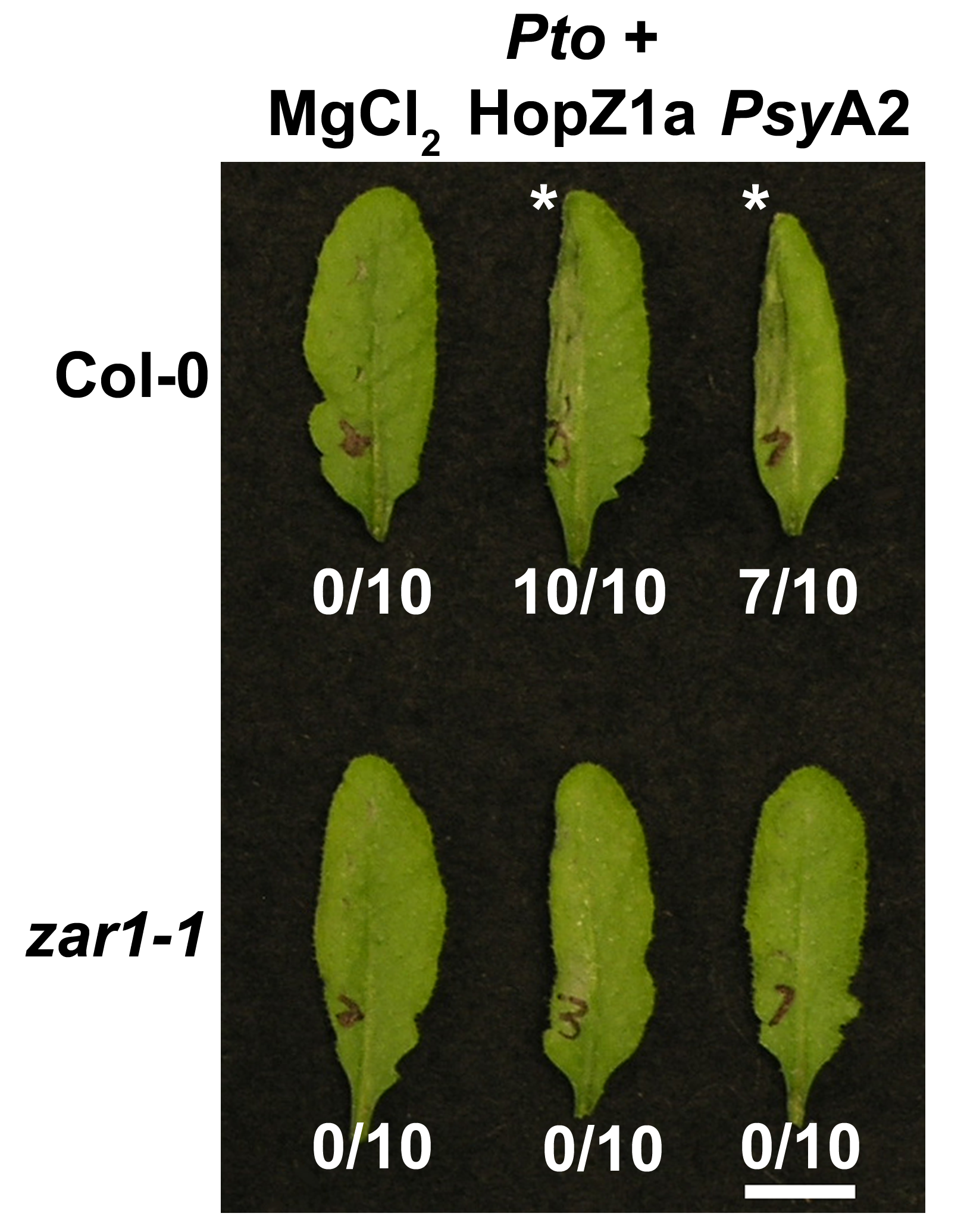

Supplement: Figure S3 — P. syringae pv. syringae strain A2 is not recognized in zar1 plants. Half-leaves of Arabidopsis Col-0 or zar1-1 plants were infiltrated with 10 mM MgCl2 or with PtoDC3000 expressing HopZ1a (Pto+HopZ1a) or PsyA2 which endogenously possesses the HopZ1a allele. The bacteria were syringe infiltrated into the leaves at 5×10^7^ cfu/mL. Photos were taken 22 hours post-infiltration. The number of leaves showing an HR is indicated below the appropriate construct. HRs are marked with an asterisk. Scale bar is 1 cm. (2.57 MB TIF) [file pgen.1000894.s003.tif]

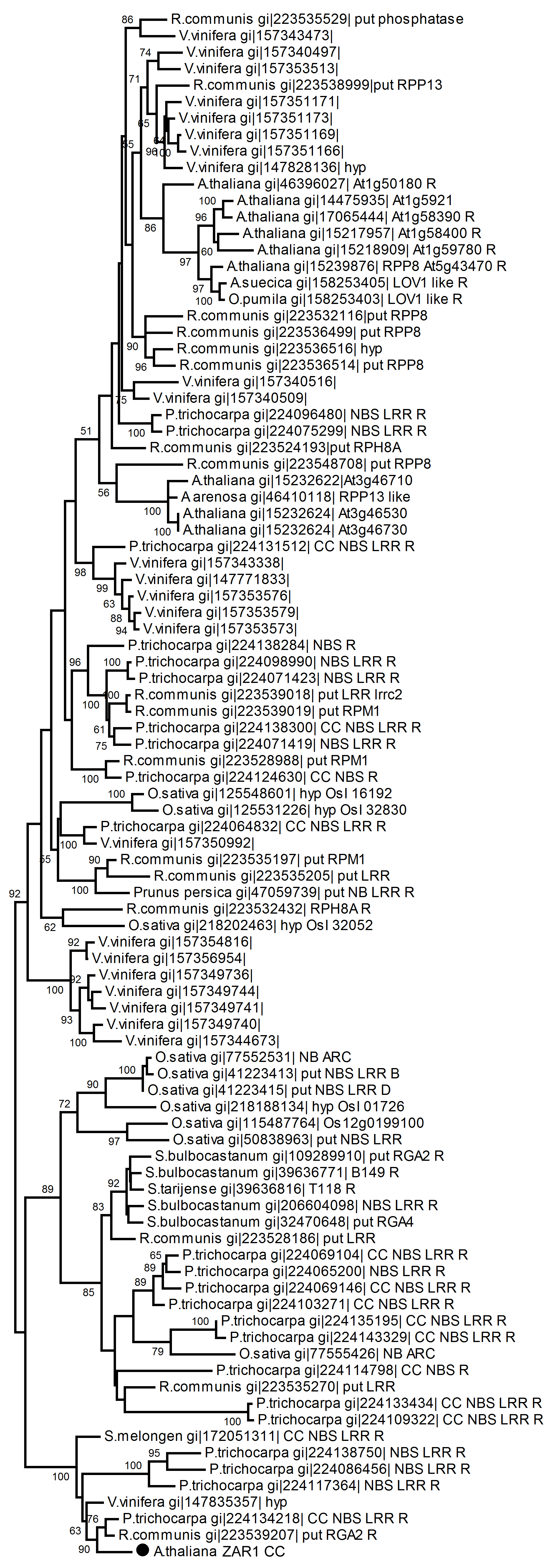

Supplement: Figure S4 — Maximum likelihood phylogenetic analysis of the coiled-coil domain from the ZAR1 protein. The tree was constructed based on a MAFFT alignment (E-INS-i algorithm) using the PALM server [75]. The best amino acid substitution model was identified by AIC criterion to be JTT+G+F, with alpha = 2.64. The initial tree was constructed using neighbor-joining, and the final tree was bootstrapped 500 times. All bootstrap scores >50 are presented above the appropriate nodes. (1.55 MB TIF) [file pgen.1000894.s004.tif]
