# Supplementary material for: Allele-Specific Virulence Attenuation of the Pseudomonas syringae HopZ1a Type III Effector via the Arabidopsis ZAR1 Resistance Protein
Source: PLoS Genet. 2010 Apr 1;6(4):e1000894. doi: 10.1371/journal.pgen.1000894 (PMC2848558; doi:10.1371/journal.pgen.1000894)
Supplement: Table S1 — Arabidopsis R Gene T–DNA Insertion Collection (ARTIC). (0.52 MB DOC) [file pgen.1000894.s005.doc]

| **Table S1.** *Arabidopsis* *R* Gene T-DNA Insertion Collection (ARTIC) | | | | | | | |
| --- | --- | --- | --- | --- | --- | --- | --- |
| **Locus** | **Other Name** | **Structure** | **Insertion Line** | **Location** | **Chr coordinates** | **Genotype** | **References** |
| At1g10920 | LOV1 | CC-NBS-LRR | SALK_152273 | Exon | 3645156-3645248 | Het | Lorang et al. 2007 |
| At1g12210 | RFL1 | CC-NBS-LRR | SALK_098988C | Exon | 4142498-4142618 | HM | Henk et al. 1999 |
| At1g12220 | RPS5 | CC-NBS-LRR | SALK_127201C | Exon | 4145965-4146308 | HM | Simonich and Innes 1995, Warren et al. 1998 |
| At1g12280 |  | CC-NBS-LRR | SALK_020392C | Exon | 4175039-4175311 | HM |  |
| At1g12290 |  | CC-NBS-LRR | SALK_012475C | Exon | 4179537-4179570 | HM |  |
| At1g15890 |  | CC-NBS-LRR | SALK_055219C | Exon | 5462119-5462422 | HM |  |
| At1g17600 |  | TIR-NBS-LRR | SALK_069931 | Exon | 6053205-6053374 | HM |  |
| At1g17610 |  | TIR-NBS | SAIL_95_C03 | Exon | 6057770-6057921 | HM |  |
| At1g17615 |  | TIR-NBS | WiscDsLox485-488F15 | 500-Promotor | 6058977-6059629 | HM |  |
|  |  | TIR-NBS | SALK_062069 | Exon | 6060640-6060775 | HM |  |
| At1g27170 |  | TIR-TIR-NBS-LRR | SALK_104245 | Exon | 9434435-9434724 | Het |  |
|  |  | TIR-TIR-NBS-LRR | SALK_125531C | 1000-promoter | 9434301-9434438 | HM |  |
| At1g27180 |  | TIR-NBS-LRR | SALK_034240C | Exon | 9443090-9443327 | HM |  |
| At1g31540 | RAC1 | TIR-NBS-LRR | GT_5_107330 | Exon | 11291232-11291703 | ND | Borhan et al. 2004 |
| At1g33560 | ADR1 | CC-NBS-LRR | SALK_114409 | 500-Promotor | 12168805-12168926 | HM | Grant et al. 2003 |
| At1g50180 |  | CC-NBS-LRR | SALK_036584 | Exon | 18588103-18588180 | Het |  |
| At1g51480 |  | CC-NBS-LRR | SALK_068767C | Exon | 19095459-19095847 | HM |  |
| At1g52660 |  | CC-NBS | SALK_001744 | 500-Promotor | 19616518-19616885 | HM |  |
| At1g53350 |  | CC-NBS-LRR | SAIL_798_B10 | 145-UTR5 | 19907539-19908202 | ND |  |
|  |  | CC-NBS-LRR | SAIL_583_B02 | Exon | 19908501-19909702 | ND |  |
| At1g56510 | WRR4 | TIR-NBS-LRR | SALK_148037C | Exon | 21173647-21173862 | HM | Borhan et al. 2008 |
| At1g56520 |  | TIR-NBS-LRR | SALK_111589C | Exon | 21180641-21180676 | HM |  |
| At1g56540 |  | TIR-NBS-LRR | SAIL_205_B06 | Exon | 21185647-21185901 | HM |  |
| At1g58390 |  | CC-NBS-LRR | SALK_054802 | Exon | 21694925-21695079 | HM |  |
| At1g58400 |  | CC-NBS-LRR | SAIL_546_E04 | Exon | 21701771-21701805 | ND |  |
| At1g58410 |  | CC-NBS-LRR | SAIL_81_F12 | Exon | 21705789-21706240 | ND |  |
|  |  | CC-NBS-LRR | SAIL_435_B03 | Exon | 21706153-21706235 | ND |  |
| At1g58602 |  | CC-NBS-LRR | SALK_045900C | 300-UTR5 | 21763777-21763856 | HM |  |
|  |  | CC-NBS-LRR | SALK_132019 | Exon | 21766496-21766689, 21766891-21766952 | ND |  |
| At1g58807 |  | CC-NBS-LRR | n/a |  |  |  |  |
| At1g58848 |  | CC-NBS-LRR | SALK_055705 | 300-UTR5 | 21791844-21792024 | ND |  |
| At1g59124 |  | CC-NBS-LRR | SAIL_194_G10 | Exon | 21820980-21821081 | Het |  |
| At1g59218 |  | CC-NBS-LRR | SALK_124243 | Exon | 21833292-21833477 | Het |  |
|  |  | CC-NBS-LRR | SALK_017424C | 145-UTR5 | 21831979-21832292 | HM |  |
| At1g59620 | CW9 | CC-NBS-LRR | SALK_024312C | Exon | 21907717-21908034 | HM |  |
| At1g59780 |  | CC-NBS-LRR | SALK_094035 | Exon | 22000993-22001067 | HM |  |
| At1g61180 |  | CC-NBS-LRR | SALK_088257 | Exon | 22555806-22556004 | Het |  |
|  |  | CC-NBS-LRR | SALK_114547 | Exon | 22556703-22556774 | Het |  |
| At1g61190 |  | CC-NBS-LRR | SALK_016752 | Exon | 22561700-22561919 | HM |  |
| At1g61300 |  | NBS-LRR^b^ | SALK_093048C | Exon | 22612240-22612522 | HM |  |
| At1g61310 |  | CC-NBS-LRR | SALK_125189C | Exon | 22617860-22617918 | HM |  |
| At1g62630 |  | CC-NBS-LRR | SALK_110355C | Exon | 23190522-23190702 | HM |  |
| At1g63350 |  | CC-NBS-LRR | SALK_025206C | Exon | 23498802-23498868 | HM |  |
| At1g63360 |  | CC-NBS-LRR | SALK_109626C | Exon | 23503888-23504150 | HM |  |
| At1g63730 |  | TIR-NBS-LRR | SALK_087810C | Exon | 23646291-23646575 | HM |  |
| At1g63740 |  | TIR-NBS-LRR | SALK_101258 | Exon | 23652268-23652381 | ND |  |
| At1g63750 |  | TIR-NBS-LRR | SALK_022493C | Exon | 23656655-23656824 | HM |  |
| At1g63860 |  | TIR-NBS-LRR | SALK_033050C | Exon | 23707202-23707504 | HM |  |
| At1g63870 |  | TIR-NBS-LRR | SAIL_452_G12 | Exon | 23710880-23710938 | ND |  |
| At1g63880 | RLM1 | TIR-NBS-LRR | SALK_110395 | Exon | 23716903-23717070 | HM | Staal et al. 2006 |
| At1g64070 | RLM1 | TIR-NBS-LRR | SALK_042846C | Exon | 23787105-23787267 | HM | Staal et al. 2006 |
| At1g65850 |  | TIR-NBS-LRR | GT_5_66042 | Exon | 24500308-24500586 | ND |  |
| At1g66090 |  | TIR-NBS | SALK_120846C | Exon | 24607897-24608199 | HM |  |
| At1g69550 |  | TIR-NBS-LRR | SALK_048285C | Exon | 26155363-26155646 | HM |  |
| At1g72840 |  | TIR-NBS-LRR | SALK_018263C | Exon | 27414190-27414244 | HM |  |
| At1g72840/50 |  | TIR-NBS-LRR | SALK_123530C | 500-Promotor | 27417460-27417840 | HM |  |
| At1g72850 |  | TIR-NBS | SALK_026014 | Exon | 27418025-27418064 | HM |  |
| At1g72860 |  | TIR-NBS-LRR | SALK_025818C | Intron | 27422712-27422767 | HM |  |
| At1g72870 |  | TIR-NBS | SALK_015695 | Exon | 27426118-27426461 | HM |  |
| At1g72890 |  | TIR-NBS | SALK_147193C | Exon | 27434749-27434925 | HM |  |
| At1g72900 |  | TIR-NBS | SALK_051714 | Exon | 27436705-27436838 | HM |  |
| At1g72910 |  | TIR-NBS | SALK_148845 | Exon | 27439989-27440197 | Het |  |
|  |  | TIR-NBS | SALK_120578C | 300-UTR3 | 27440354-27440653 | HM |  |
| At1g72940 |  | TIR-NBS | SALK_109730 | Exon | 27446636-27446842 | HM |  |
| At1g72950 |  | TIR-NBS | SALK_151624 | 145-UTR5 | 27448103-27448209 | Het |  |
| At2g14080 | RPP28 | TIR-NBS-LRR | SALK_105230C | Intron | 5933103-5933228 | HM |  |
| At2g16870 |  | TIR-NBS-LRR | SALK_145219C | Exon | 7318371-7318685 | HM |  |
| At2g17050 |  | TIR-NBS-LRR-TIR | SALK_138745 | Exon | 7420896-7421066 | HM |  |
| At2g17060 |  | TIR-NBS-LRR | SALK_021169C | Exon | 7430975-7431628 | HM |  |
| At3g04210 |  | TIR-NBS | SALK_044806 | Exon | 1107048-1107214 | HM |  |
| At3g04220 |  | TIR-NBS-LRR | GT_5_106122 | Exon | 1111002-1111211 | ND |  |
| At3g07040 | RPM1 | CC-NBS-LRR | SALK_146601C | Exon | 2228857-2229233 | HM | Dangl et al. 1992, Kiedrowski et al. 1992, Grant et al. 1995, |
| At3g14460 |  | CC-NBS-LRR | SALK_138613C | Exon | 4853979-4854271 | HM |  |
| At3g14470 |  | CC-NBS-LRR | SALK_077805 | Exon | 4858716-4858804 | HM |  |
| At3g15700 |  | CC-NBS | n/a |  |  |  |  |
| At3g25510 |  | TIR-NBS-LRR | SALK_032836C | Intron | 9267461-9267611 | HM |  |
| At3g44400 |  | TIR-NBS-LRR | SALK_045528C | Exon | 16057629-16058090 | HM |  |
| At3g44480 | RPP1 | TIR-NBS-LRR | SALK_019847C | Intron | 16102055-16102380 | HM | Botella et al. 1998 |
| At3g44630 |  | TIR-NBS-LRR | SALK_144159C | Exon | 16206912-16207346 | HM |  |
| At3g44670 | RPP1-like | TIR-NBS-LRR | SALK_029707 | Exon | 16230493-16230560 | Het | Botella et al. 1998 |
| At3g46530 | RPP13 | CC-NBS-LRR | SAIL_1175_H09 | Exon | 17143790-17144112 | Het | Bittner-Eddy et al. 2000 |
| At3g46710 |  | CC-NBS-LRR | SALK_102235C | Exon | 17219377-17219494 | HM |  |
| At3g46730 |  | CC-NBS-LRR | SAIL_35_F09 | Exon | 17225932-17226338 | Het |  |
| At3g50950 | ZAR1 | CC-NBS-LRR | SALK_013297C | Exon | 18949010-18949383 | HM | this pub |
| At3g51560 |  | TIR-NBS-LRR | SALK_026635C | Exon | 19136113-19136521 | HM |  |
| At3g51570 |  | TIR-NBS-LRR | SALK_139528C | Exon | 19137434-19137649 | HM |  |
| At4g08450 |  | TIR-NBS-LRR | SALK_091199C | 500-Promotor | 5365239-5365453 | HM |  |
| At4g09360 |  | NBS-LRR^c^ | SALK_147487 | Exon | 5942943-5943208 | Het |  |
| At4g09420 |  | TIR-NBS | SALK_057795 | Exon | 5962731-5963069 | Het |  |
|  |  | TIR-NBS | SALK_056031 | Exon | 5962108-5962413 | ND |  |
| At4g09430 |  | TIR-NBS-LRR | SALK_074272C | Intron | 5975154-5975254 | HM |  |
| At4g10780 |  | CC-NBS-LRR | SALK_066132 | Exon | 6636905-6637015 | Het |  |
| At4g11170 |  | TIR-NBS-LRR | SALK_091592C | Exon | 6816768-6817020 | HM |  |
| At4g12010 |  | TIR-NBS-LRR | SALK_145043C | Exon | 7197593-7197676 | HM |  |
| At4g12020 | WRKY19, MAPKKK11 | W-TIR-NBS-LRR-M | SALK_086102C | Exon | 7207087-7207347 | HM |  |
| At4g14370 |  | TIR-NBS-LRR | SAIL_753_B02 | Exon | 8282175-8282732 | HM |  |
| At4g14610^a^ |  | CC-NBS-LRR | SALK_080562C | Exon | 8381111-8381477 | HM |  |
| At4g16860 | RPP4 | TIR-NBS-LRR | SAIL_559_B03 | Exon | 9488654-9489156 | ND | van der Biezen et al. 2002 |
|  | RPP4 | TIR-NBS-LRR | SAIL_664_E06 | 500-Promotor | 9495874-9496079 | ND |  |
| At4g16890 | SNC1 | TIR-NBS-LRR | SALK_047058C | Exon | 9505038-9505426 | HM | Li et al. 2001 |
| At4g16900 |  | TIR-NBS-LRR | SALK_034491 | Exon | 9514999-9515337 | Het |  |
|  |  | TIR-NBS-LRR | SALK_130978C | 145-UTR5 | 9516692-9516875 | HM |  |
| At4g16920 |  | TIR-NBS-LRR | SALK_073988 | Exon | 9525677-9526374 | HM |  |
| At4g16940 |  | TIR-NBS-LRR | SALK_032697C | Exon | 9535509-9535656 | HM |  |
| At4g16950 | RPP5 | TIR-NBS-LRR | SALK_028077 | Exon | 9544187-9544311 | HM | Parker et al. 1997 |
| At4g16960 |  | TIR-NBS-LRR | SALK_044486 | Exon | 9550863-9550975 | Het |  |
|  |  | TIR-NBS-LRR | SALK_126120 | Exon | 9550564-9550975 | ND |  |
| At4g16990 | RLM3 | TIR-NBS | SALK_129999C | Intron | 9564011-9564375 | HM | Staal et al. 2008 |
|  | RLM3 | TIR-NBS | SALK_037233 | 300-UTR3 | 9565510-9565644 | HM |  |
| At4g19050 |  | Divergent NBS-LRR | SALK_077443C | Exon | 10440472-10440605 | HM |  |
| At4g19060 |  | CC-NBS | n/a |  |  |  |  |
| At4g19500 | RPP2A | TIR-NBS-TIR-NBS-LRR | SALK_078316 | Exon | 10629942-10630015 | HM | Sinapidou et al. 2004 |
| At4g19510 | RPP2B | TIR-NBS-LRR | SALK_030554 | Intron | 10637384-10637586 | ND | Sinapidou et al. 2004 |
| At4g19520 |  | TIR-NBS-LRR-TIR | SALK_118667C | Exon | 10644560-10644732 | HM |  |
| At4g19530 | N | TIR-NBS-LRR | SALK_026650C | Exon | 10655823-10656041 | HM | Whitham et al. 1994 |
| At4g23440 |  | X-TIR-NBS-X | SAIL_882_B08 | Exon | 12237810-12238601 | HM |  |
| At4g26090 | RPS2 | CC-NBS-LRR | SALK_087581 | Exon | 13225456-13225687 | HM | Bent et al. 1994, Mindrinos et al. 1994 |
| At4g27190 |  | CC-NBS-LRR | SALK_127153C | Exon | 13621579-13621811 | HM |  |
| At4g27220 |  | CC-NBS-LRR | SALK_127692C | Exon | 13634341-13634737 | HM |  |
| At4g33300 | ADR1-like1 | CC-NBS-LRR | SAIL_302_C06 | Exon | 16053307-16053517 | HM | Grant et al. 2003 |
| At4g36140 |  | TIR-NBS-TIR-NBS-LRR | SALK_126390C | Exon | 17099323-17099521 | HM |  |
| At4g36150 |  | TIR-NBS-LRR | SALK_084909C | Exon | 17107462-17107900 | HM |  |
| At5g04720 | ADR1-like2 | CC-NBS-LRR | SALK_126422C | Exon | 1360807-1361136 | HM | Grant et al. 2003 |
| At5g05400 |  | CC-NBS-LRR | SALK_005464C | Exon | 1597820-1597975 | HM |  |
| At5g11250 |  | TIR-NBS-LRR | SALK_065253C | Exon | 3590102-3590339 | HM |  |
| At5g17680 |  | TIR-NBS-LRR | SALK_004241C | Exon | 5824929-5825216 | HM |  |
| At5g17890 | DAR4 | TIR-NBS-LRR-X | SALK_024264 | Exon | 5918046-5918134 | HM |  |
| At5g17970 |  | TIR-NBS-LRR | SALK_005505C | Exon | 5949929-5950213 | HM |  |
| At5g18350 |  | TIR-NBS-LRR | SALK_135943 | Exon | 6077658-6077960 | HM |  |
| At5g18360 |  | TIR-NBS-LRR | SALK_088169C | Exon | 6080268-6080708 | HM |  |
| At5g18370 |  | TIR-NBS-LRR | SALK_096162 | Exon | 6085798-6086175 | HM |  |
| At5g22690 |  | TIR-NBS-LRR | SALK_061751C | Exon | 7541599-7541631 | HM |  |
| At5g35450 |  | CC-NBS-LRR | SALK_000281C | Exon | 13687407-13687790 | HM |  |
| At5g36930 |  | TIR-NBS-LRR | SALK_084173C | Exon | 14586412-14586713 | HM |  |
| At5g38340 |  | TIR-NBS-LRR | SALK_130839 | Exon | 15340181-15340238 | HM |  |
| At5g38344 |  | TIR-NBS-LRR | SALK_016958C | Exon | 15326471-15326711 | HM |  |
| At5g38350 |  | TIR-NBS-LRR | SALK_104727C | Exon | 15347862-15348310 | HM |  |
| At5g38850 |  | TIR-NBS-LRR | SALK_134889C | Exon | 15556851-15557157 | HM |  |
| At5g40060 |  | TIR-NBS-LRR | SALK_127539C | Exon | 16053996-16054313 | HM |  |
| At5g40090 |  | TIR-NBS | SAIL_558_A07 | Exon | 16059826-16059968 | Het |  |
| At5g40100 |  | TIR-NBS-LRR | SAIL_743_E07 | Exon | 16063512-16064130 | HM |  |
| At5g40910 |  | TIR-NBS-LRR | SALK_043422C | Exon | 16413097-16413157 | HM |  |
| At5g40920^a^ |  | TIR-NBS-LRR | SALK_009018C | Exon | 16420595-16420839 | HM |  |
| At5g41540 |  | TIR-NBS-LRR | SALK_034471C | Exon | 16631798-16631989 | HM |  |
| At5g41550 |  | TIR-NBS-LRR | SALK_040476C | Exon | 16636619-16637037 | HM |  |
| At5g41740 | SSI4-like^d^ | TIR-NBS-LRR | GT_5_109127 | Exon | 16707467-16712800 | ND | Shirano et al. 2002 |
| At5g41750 | SSI4-like^d^ | TIR-NBS-LRR | SALK_133292 | Exon | 16713981-16714408 | ND | Shirano et al. 2002 |
|  |  | TIR-NBS-LRR | SAIL_861_H05 | Exon | 16712907-16713195 | ND |  |
| At5g43470 | RPP8, HRT, RCY1 | CC-NBS-LRR | SALK_070397 | Exon | 17483481-17483850 | HM | McDowell et al. 1998, Cooley et al. 2000, Takahashi et al. 2002 |
| At5g43730 |  | CC-NBS-LRR | SALK_075439 | Exon | 17579162-17579263 | HM |  |
| At5g43740 |  | CC-NBS-LRR | SALK_025605C | Exon | 17583242-17583484 | HM |  |
| At5g44510 | TAO1 | TIR-NBS-LRR | SALK_124245C | Exon | 17950584-17950912 | HM | Eitas et al. 2008 |
| At5g44870 |  | TIR-NBS-LRR | SALK_068316C | 500-Promotor | 18131473-18131634 | HM |  |
|  |  | TIR-X(TX) | SALK_004032C | 145-UTR5 | 18154482-18154914 | HM |  |
| At5g45050 | WRKY16, TTR1 | TIR-NBS-LRR-W | SALK_095020C | Exon | 18198584-18198683 | HM | Lee et al. 1996 |
| At5g45060 |  | TIR-NBS-LRR | SALK_063382 | Exon | 18201877-18202119 | HM |  |
| At5g45200 |  | TIR-NBS-LRR | SALK_149876 | Exon | 18301729-18302154 | ND |  |
| At5g45210 |  | TIR-NBS-LRR | SALK_064488 | Exon | 18314832-18314892 | ND |  |
| At5g45230 |  | TIR-NBS-LRR | SALK_069384C | Exon | 18319933-18320088 | HM |  |
| At5g45240 |  | NBS-LRR^c^ | SALK_034517C | Exon | 18331036-18331078 | HM |  |
| At5g45250 | RPS4, RCH2 | TIR-NBS-LRR | SALK_057697C | Exon | 18341662-18341833 | HM | Gassmann et al. 1999, Birker et al. 2009, Narusaka et al. 2009 |
| At5g45260 | RRS1, WRKY52, SLH1, RCH2 | TIR-NBS-LRR | SALK_061602 | Exon | 18346635-18346709 | Het | Deslandes et al. 2002, Noutoshi et al. 2005, Birker et al. 2009, Narusaka et al. 2009, |
| At5g45440 |  | NBS only^b^ | SALK_023316C | Exon | 18429609-18429794 | HM |  |
| At5g45490 |  | CC-NBS | SALK_007202C | 500-Promotor | 18447741-18448142 | HM |  |
| At5g45510 |  | Divergent NBS-LRR | SAIL_335_H01 | Exon | 18462735-18463259 | Het |  |
| At5g46260 |  | TIR-NBS-LRR | SALK_005326 | 300-UTR3 | 18775837-18776144 | HM |  |
| At5g46270 |  | TIR-NBS-LRR | SAIL_508_E02 | Exon | 18784555-18784883 | Het |  |
| At5g46450 |  | TIR-NBS-LRR | SALK_058343 | Exon | 18854043-18854156 | HM |  |
|  |  | TIR-NBS-LRR | SALK_054265C | Exon | 18856596-18856783 | HM |  |
| At5g46470 | RPS6 | TIR-NBS-LRR | SALK_029541 | Exon | 18860660-18861088 | Het | Kim et al. 2009 |
| At5g46490 |  | TIR-NBS-LRR | SAIL_836_H10 | 1000-promoter | 18867479-18867541 | HM |  |
| At5g46510 |  | TIR-NBS-LRR | SAIL_402_G03 | 500-Promotor | 18877335-18877519 | HM |  |
| At5g46520 |  | TIR-NBS-LRR | SALK_097845 | Exon | 18887009-18887422 | Het |  |
| At5g47250 |  | CC-NBS-LRR | SAIL_134_E12 | Exon | 19204579-19204664 | ND |  |
|  |  | CC-NBS-LRR | SAIL_134_D12 | Exon | 19204579-19204667 | ND |  |
| At5g47260 |  | CC-NBS-LRR | SALK_095083 | Exon | 19207827-19208016 | HM |  |
| At5g47280 | ADR1-like3 | NBS-LRR^b^ | SAIL_757_H10 | Exon | 19212524-19212671 | Het | Grant et al. 2003 |
| At5g48620 |  | CC-NBS-LRR | SALK_034883 | 500-Promotor | 19733877-19734164 | Het |  |
| At5g48770 |  | TIR-NBS-LRR | SALK_103905C | Exon | 19794074-19794186 | HM |  |
| At5g48780 |  | TIR-NBS | SALK_152056 | 3'UTR | 19796706-19796854 | HM |  |
| At5g49140 |  | TIR-NBS-LRR | SALK_088388C | Exon | 19940191-19940663 | HM |  |
| At5g51630 |  | TIR-NBS-LRR | SALK_003417C | Intron | 20989975-20990112 | HM |  |
| At5g56220 |  | X-TIR-NBS-X | SALK_132425C | Exon | 22771851-22772266 | HM |  |
| At5g58120 |  | TIR-NBS-LRR | SALK_061294 | Exon | 23534763-23534958 | ND |  |
| At5g63020 |  | CC-NBS-LRR | SAIL_885_C06 | Exon | 25302443-25302939 | HM |  |
| At5g66630 | DAR5 | CC-NBS-X | SALK_070867C | Exon | 26612449-26612524 | HM |  |
| At5g66900 |  | CC-NBS-LRR | SALK_105768 | Exon | 26734453-26734561 | ND |  |
| At5g66910 |  | CC-NBS-LRR | SALK_020974 | Exon | 26737274-26737691 | Het |  |

^a^ pseudogene

^b^ phylogenetic analysis shows that it groups with CC-NBS-LRR class R proteins (Meyers et al. 2003)

^c^ phylogenetic analysis shows that it groups with TIR-NBS-LRR class R proteins (Meyers et al. 2003)

^d^ SSI4 was first identified in the Nossen ecotype of *Arabidopsis thaliana*. BLAST was used to identify SSI4-like genes in Col-0.

Abbreviations in table:

CC: coiled-coil

Het: heterozygous

HM: homozygous

M: MAPK

NBS: nucleotide-binding-site

ND: not determined

LRR: leucine-rich-repeat

TIR: Toll interleukin 1 receptor domain

UTR3: 3’ untranslated region

UTR5: 5’ untranslated region

W: WRKY domain

X: other domain

References

Bent, A.F., Kunkel, B.N., Dahlbeck, D., Brown, K.L., Schmidt, R., et al. (1994) *RPS2* of *Arabidopsis thaliana*: a leucine-rich repeat class of plant disease resistance genes*.* Science 265: 1856-1860.

Birker, D., Heidrich, K., Takahara, H., Narusaka, M., Deslandes, L., et al. (2009) A locus conferring resistance to *Colletotrichum higginsianum* is shared by four geographically distinct *Arabidopsis* accessions*.* Plant J. 60: 602-613.

Bittner-Eddy, P.D., Crute, I.R., Holub, E.B., and Beynon, J.L. (2000) *RPP13* is a simple locus in *Arabidopsis thaliana* for alleles that specify downy mildew resistance to different avirulence determinants in *Peronospora parasitica.* Plant J. 21: 177-188.

Borhan, M.H., Gunn, N., Cooper, A., Gulden, S., Tor, M., et al. (2008) WRR4 encodes a TIR-NB-LRR protein that confers broad-spectrum white rust resistance in *Arabidopsis thaliana* to four physiological races of *Albugo candida.* Mol. Plant-Microbe Interact. 21: 757-768.

Borhan, M.H., Holub, E.B., Beynon, J.L., Rozwadowski, K., and Rimmer, S.R. (2004) The *Arabidopsis* TIR-NB-LRR gene *RAC1* confers resistance to *Albugo candida* (white rust) and is dependent on EDS1 but not PAD4*.* Mol. Plant-Microbe Interact. 17: 711-719.

Botella, M.A., Parker, J.E., Frost, L.N., Bittner-Eddy, P.D., Beynon, J.L., et al. (1998) Three genes of the *Arabidopsis* *RPP1* complex resistance locus recognize distinct *Peronospora parasitica* avirulence determinants*.* Plant Cell 10: 1847-1860.

Cooley, M.B., Pathirana, S., Wu, H.J., Kachroo, P., and Klessig, D.F. (2000) Members of the *Arabidopsis* *HRT/RPP8* family of resistance genes confer resistance to both viral and oomycete pathogens*.* Plant Cell 12: 663-676.

Dangl, J.L., Ritter, C., Gibbon, M.J., Mur, L.A.J., Wood, J.R., et al. (1992) Functional homologs of the *Arabidopsis* *RPM1* disease resistance gene in bean and pea*.* Plant Cell 4: 1359-1369.

Deslandes, L., Olivier, J., Theulieres, F., Hirsch, J., Feng, D.X., et al. (2002) Resistance to *Ralstonia solanacearum* in *Arabidopsis thaliana* is conferred by the recessive *RRS1-R* gene, a member of a novel family of resistance genes*.* Proc. Natl. Acad. Sci. U. S. A. 99: 2404-2409.

Eitas, T.K., Nimchukt, Z.L., and Dangl, J.L. (2008) *Arabidopsis* TAO1 is a TIR-NB-LRR protein that contributes to disease resistance induced by the *Pseudomonas syringae* effector AvrB*.* Proc. Natl. Acad. Sci. U. S. A. 105: 6475-6480.

Gassmann, W., Hinsch, M.E., and Staskawicz, B.J. (1999) The *Arabidopsis* *RPS4* bacterial resistance gene is a member of the TIR-NBS-LRR family of disease resistance genes*.* Plant J. 20: 265-277.

Grant, J.J., Chini, A., Basu, D., and Loake, G.J. (2003) Targeted activation tagging of the *Arabidopsis* NBS-LRR gene, *ADR1*, conveys resistance to virulent pathogens*.* Mol. Plant-Microbe Interact. 16: 669-680.

Grant, M.R., Godiard, L., Straube, E., Ashfield, T., Lewald, J., et al. (1995) Structure of the *Arabidopsis* *RPM1* gene enabling dual-specificity disease resistance*.* Science 269: 843-846.

Henk, A.D., Warren, R.F., and Innes, R.W. (1999) A new Ac-like transposon of *Arabidopsis* is associated with a deletion of the *RPS5* disease resistance gene*.* Genetics 151: 1581-1589.

Kiedrowski, S., Kawalleck, P., Hahlbrock, K., Somssich, I.E., and Dangl, J.L. (1992) Rapid activation of a novel plant defense gene is strictly dependent on the *Arabidopsis* *RPM1* disease resistance locus*.* EMBO J. 11: 4677-4684.

Kim, S.H., Kwon, S.I., Saha, D., Anyanwu, N.C., and Gassmann, W. (2009) Resistance to *Pseudomonas syringae* effector HopA1 is governed by the TIR-NBS-LRR protein RPS6 and is enhanced by mutations in *SRFR1.* Plant Physiol. 150: 1723-1732.

Lee, J.M., Hartman, G.L., Domier, L.L., and Bent, A.F. (1996) Identification and map location of *TTR1*, a single locus in *Arabidopsis thaliana* that confers tolerance to tobacco ringspot nepovirus*.* Mol. Plant-Microbe Interact. 9: 729-735.

Li, X., Clarke, J.D., Zhang, Y.L., and Dong, X.N. (2001) Activation of an EDS1-mediated *R* gene pathway in the *snc1* mutant leads to constitutive, NPR1-independent pathogen resistance*.* Mol. Plant-Microbe Interact. 14: 1131-1139.

Lorang, J.M., Sweat, T.A., and Wolpert, T.J. (2007) Plant disease susceptibility conferred by a "resistance" gene*.* Proc. Natl. Acad. Sci. U. S. A. 104: 14861-14866.

McDowell, J.M., Dhandaydham, M., Long, T.A., Aarts, M.G.M., Goff, S., et al. (1998) Intragenic recombination and diversifying selection contribute to the evolution of downy mildew resistance at the *RPP8* locus of *Arabidopsis.* Plant Cell 10: 1861-1874.

Meyers, B.C., Kozik, A., Griego, A., Kuang, H.H., and Michelmore, R.W. (2003) Genome-wide analysis of NBS-LRR-encoding genes in *Arabidopsis.* Plant Cell 15: 809-834.

Mindrinos, M., Katagiri, F., Yu, G.L., and Ausubel, F.M. (1994) The *A. thaliana* disease resistance gene *RPS2* encodes a protein containing a nucleotide-binding site and leucine-rich repeats*.* Cell 78: 1089-1099.

Narusaka, M., Shirasu, K., Noutoshi, Y., Kubo, Y., Shiraishi, T., et al. (2009) *RRS1* and *RPS4* provide dual *Resistance*-gene system against fungal and bacterial pathogens*.* Plant J. 60: 218-226.

Noutoshi, Y., Ito, T., Seki, M., Nakashita, H., Yoshida, S., et al. (2005) A single amino acid insertion in the WRKY domain of the *Arabidopsis* TIR-NBS-LRR-WRKY-type disease resistance protein *SLH1* (sensitive to low humidity 1) causes activation of defense responses and hypersensitive cell death*.* Plant J. 43: 873-888.

Parker, J.E., Coleman, M.J., Szabo, V., Frost, L.N., Schmidt, R., et al. (1997) The *Arabidopsis* downy mildew resistance gene *RPP5* shares similarity to the Toll and interleukin-1 receptors with N and L6*.* Plant Cell 9: 879-894.

Shirano, Y., Kachroo, P., Shah, J., and Klessig, D.F. (2002) A gain-of-function mutation in an *Arabidopsis* Toll Interleukin-1 Receptor-Nucleotide Binding Site-Leucine-Rich Repeat type *R* gene triggers defense responses and results in enhanced disease resistance*.* Plant Cell 14: 3149-3162.

Simonich, M.T. and Innes, R.W. (1995) A disease resistance gene in *Arabidopsis* with specificity for the *avrPph3* gene of *Pseudomonas syringae* pv. phaseolicola*.* Mol. Plant-Microbe Interact. 8: 637-640.

Sinapidou, E., Williams, K., Nott, L., Bahkt, S., Tor, M., et al. (2004) Two TIR-NB-LRR genes are required to specify resistance to *Peronospora parasitica* isolate Cala2 in *Arabidopsis.* Plant J. 38: 898-909.

Staal, J., Kaliff, M., Bohman, S., and Dixelius, C. (2006) Transgressive segregation reveals two *Arabidopsis* TIR-NB-LRR resistance genes effective against *Leptosphaeria maculans*, causal agent of blackleg disease*.* Plant J. 46: 218-230.

Staal, J., Kaliff, M., Dewaele, E., Persson, M., and Dixelius, C. (2008) *RLM3*, a TIR domain encoding gene involved in broad-range immunity of *Arabidopsis* to necrotrophic fungal pathogens*.* Plant J. 55: 188-200.

Takahashi, H., Miller, J., Nozaki, Y., Sukamto, Takeda, M., et al. (2002) *RCY1*, an *Arabidopsis thaliana* *RPP8/HRT* family resistance gene, conferring resistance to cucumber mosaic virus requires salicylic acid, ethylene and a novel signal transduction mechanism*.* Plant J. 32: 655-667.

van der Biezen, E.A., Freddie, C.T., Kahn, K., Parker, J.E., and Jones, J.D.G. (2002) *Arabidopsis* *RPP4* is a member of the *RPP5* multigene family of TIR-NB-LRR genes and confers downy mildew resistance through multiple signalling components*.* Plant J. 29: 439-451.

Warren, R.F., Henk, A., Mowery, P., Holub, E., and Innes, R.W. (1998) A mutation within the leucine-rich repeat domain of the *Arabidopsis* disease resistance gene *RPS5* partially suppresses multiple bacterial and downy mildew resistance genes*.* Plant Cell 10: 1439-1452.

Whitham, S., Dinesh-Kumar, S.P., Choi, D., Hehl, R., Corr, C., et al. (1994) The product of the Tobacco Mosaic Virus resistance gene *N*: similarity to Toll and the interleukin-1 receptor*.* Cell 78: 1101-1115.
